# Chronotropic incompetence and a higher frequency of myocardial ischemia in exercise echocardiography

**DOI:** 10.1186/1476-7120-5-38

**Published:** 2007-11-02

**Authors:** Joselina LM Oliveira, Thiago JS Góes, Thaiana A Santana, Thiago F Travassos, Lívia D Teles, Fernando D Anjos-Andrade, Adão C Nascimento-Júnior, Érica O Alves, Martha A Barreto, José A Barreto-Filho, Argemiro D'Oliveira, Antônio CS Sousa

**Affiliations:** 1 Department of Internal Medicine, Cardiology Division, Federal University of Sergipe, Aracaju, Sergipe, Brazil; 2 Laboratory of Echocardiography of the São Lucas Hospital, Aracaju, Sergipe, Brazil; 3 Department of Internal Medicine, School of Medicine, Federal University of the Bahia, Salvador, Bahia, Brazil

## Abstract

**Background:**

Exercise echocardiography (EE) is an established method to diagnose coronary artery disease (CAD). Chronotropic incompetence (CI) during the EE may be a marker of myocardial ischemia. The purpose of this investigation was to evaluate the additive value of CI during EE in CAD diagnosis.

**Methods:**

Between 2000 and 2006, 4042 patients (1900 men with a mean age of 56 ± 11 years) were evaluated by EE. Based on the heart rate (HR) reached during the exercise test, the subjects were divided into two groups: G1 group – 490 patients who failed to achieve 85% of the maximal age-predicted HR, and G2 group – 3552 patients who were able to achieve 85% of the maximal age-predicted HR.

Clinical characteristics, left ventricular wall motion abnormalities – wall motion score index (WMSI) – and coronary angiography (CA) were the parameters compared between the two groups.

**Results:**

The left ventricular wall motion abnormalities were more frequent in G1 group than in G2 group (54% *versus *26%; P < 0.00001). WMSI was higher in G1 group than in G2 group, both at rest (1.06 ± 0.17 *versus *1.02 ± 0.09; P < 0.0001) and after exercise (1.12 ± 0.23 *versus *1.04 ± 0.21; P < 0.0001).

In G1 group, 82% of the patients with positive EE for myocardial ischemia presented obstructive coronary, compared to 71% (P = 0.03) in G2 group.

**Conclusion:**

CI is associated with a higher frequency of myocardial ischemia during EE, reinforcing the concept that CI is a marker of the severity of myocardial ischemia.

## Background

Chronotropic incompetence (CI), characterized by an attenuated heart rate (HR) response to exercise, was defined as the failure to achieve 85% of the maximum age-predicted HR [1] and it is not an uncommon finding during treadmill exercise. Although the underlying mechanism is not well defined, it represents an independent predictor of mortality and incidence of CAD [2,3]. The reasons for such an association are not properly explained, although several mechanisms have been proposed, such as: severity of CAD, left ventricular (LV) dilation, parasympathetic hyperactivity, sinus node dysfunction, ischemia and advanced age [4,5].

A recent research analyzed endothelial vasodilator function, inflammatory markers and N-terminal pro-brain natriuretic peptide on patients with and without chronotropic incompetence to exercise test and concluded that patients with impaired chronotropic response to graded exercise had endothelial dysfunction, enhanced systemic inflammation and higher N-terminal pro-brain natriuretic peptide concentrations. These findings may partly explain the mechanism of chronotropic incompetence as a predictor of cardiovascular risk and increased mortality [6].

It has been shown that CI is an independent predictor of death, even in patients taking drugs that interfere with chronotropism such as beta blockers [7].

The treadmill exercise test (ET) is a noninvasive method and one of the most recommended methods for diagnostic and prognostic evaluation of CAD [8]. However, in clinical practice, CI may limit the value of treadmill exercise to confirm CAD diagnosis which is based on ST segment changes occurring at higher rate [9].

Exercise echocardiography (EE) is a noninvasive method well established for the diagnosis and risk stratification of CAD, especially when left bundle branch block (LBBB), left ventricular overload and pre-excitation syndrome are present [10]. The imbalance between oxygen supply and demand leads to myocardial changes caused by ischemia. These alterations occur in a time sequence of pathophysiological phenomena described by Heyndrickx et al and called "ischemia cascade", which is temporally characterized by heterogeneous perfusion, metabolic changes, diastolic dysfunction, regional dyskinesia, electrocardiographic changes and angina [11]. Therefore, EE is able to detect ischemia alterations earlier than ET. However, the role of EE in patients who fail to achieve 85% of the maximum age-predicted HR remains not fully explained. We hypothesize that EE may bring a significant contribution to this subgroup of patients, since it is more sensitive than conventional ET and it is also able to detect resting segmental LV changes.

The purpose of the present investigation is to assess the value of exercise echocardiography in diagnosis of coronary artery disease in patients with chronotropic incompetence, considering coronary angiography (CA) as the golden standard.

## Methods

### Patient population

From December 2000 to November 2006, 4042 consecutive patients with known or suspected coronary artery disease were referred to the Laboratory of Echocardiography at São Lucas Hospital (city of Aracaju, State of Sergipe, Brazil) to undergo EE. The ethical principles that guide human experimentation were carefully observed and informed consent was obtained from all the patients involved in the research. The study was approved by the Ethics and Research Committee of the Federal University of Sergipe.

Exclusion criteria were refusal to participate in the research (12 patients), poor imaging quality (80 patients), atrial fibrillation (5 patients) and significant valvular heart disease (15 patients).

For the analysis, patients were divided into two groups: G1 group – 490 patients who failed to achieve 85% of the maximal age-predicted HR and G2 group – 3552 patients who were able to achieve 85% of the maximal age-predicted HR.

The presence or absence of symptoms such as typical or atypical angina, risk factors of CAD and the use of medication were registered. Hypercholesterolemia was defined as serum total cholesterol levels higher than 200 mg/dl (after a 12-hour fasting) and hypertriglyceridemia as serum triglycerides levels higher than 150 mg/dl (after a 12-hour fasting) or by the use of lipid-lowering agents (vastatins and/or fibrates). Systemic hypertension was considered when blood pressure measurements on upper limb, at rest, were ≥ 140 × 90 mmHg or by the use of anti-hypertensive medication. Diabetes mellitus was defined as fasting glucose levels above of 126 mg/dl or by the use of insulin or oral hypoglycemic agents. Previousmyocardium infarction was defined based on clinical history and/or complementary examinations such as electrocardiogram, echocardiogram and coronary angiography.

Single or combined indications for EE were: assessment of chest pain, preoperative assessment of cardiac risk for non-cardiac surgery, presence of a positive ET for myocardial ischemia in patients without clinical probability of CAD, negative ET for myocardial ischemia in patients with clinical probability of CAD, stratification of an already established CAD and risk stratification after myocardial infarction.

All the patients were examined after having a light meal. On the day of the exam, they avoided any excessive physical activity and beta blocker had been discontinued four days before the test. All the investigation was conducted with the individual breathing spontaneously in room air, at a constant room temperature of approximately 20°C to 24°C.

The study consisted of the performance of a complete clinical investigation (history taking and physical examination) followed by a 12-lead electrocardiogram (ECG) and resting echocardiogram. Then, patients underwent exercise treadmill and immediately, another echocardiogram was obtained. CA was indicated for the patients who presented a positive EE for myocardial ischemia.

### Exercise echocardiography protocol

All patients underwent symptom-limited treadmill exercise testing according to the standard Bruce protocol. Heart rate was continuously recorded, and patients were strongly encouraged to reach > 85% of maximal age-predicted heart rate. Workload was expressed in metabolic equivalents (METS). The exercise was interrupted whenever the maximum age-predicted HR was exceeded or in the presence of the following signs and/or symptoms: chest pain, shortness of breath, muscle fatigue, hypertension (blood pressure ≥ 220 × 120 mmHg), hypotension, presyncope and severe arrhythmias. During the test, the individuals were continuously monitored with a three-lead ECG.

ET was considered positive for myocardial ischemia if there was a horizontal or down-sloping ST-segment depression, of ≥ 1 mm for men and 1.5 mm for women, at 80 ms after the J point. In the presence of electrocardiographic changes which were suggestive of the left bundle branch block (LBBB), left ventricular hypertrophy, pre-excitation syndrome and use of medication (digitalis), the test was considered non-diagnostic [8].

The echocardiographs were performed with Hewlett-Packard/Phillips SONOS 5500 systems. Two-dimensional echocardiograph images were obtained from the parasternal and apical windows at rest and immediately after exercise. Both digitized and videotape-recorded or digital video display (DVD) were used for the interpretation of the studies [12]. Regional wall motion was assessed semi quantitatively by experienced echocardiographers, with level III training, as recommended by the American Society of Echocardiography. Wall motion at rest and with exercise was scored 1 through 5 (1 = normal) according to a 16-segment model [13]. Wall motion score index (WMSI), was determined at rest and peak exercise as the sum of the segmental scores divided by the number of visualized segments. The development of new or worsening wall motion was considered indicative of myocardial ischemia. A wall motion abnormality present at rest and unchanged with exercise was classified as "fixed". Therefore, exercise echocardiography results were defined as abnormal if there was ischemia or fixed wall motion abnormalities [14]. We considered EE fixed ischemia and induced ischemia when WSMI became worse after exercise in patients with previous alterations.

### Coronary angiography (CA)

CA was performed voluntarily in the patients who had a positive EE for myocardial ischemia, using the Judkins technique, preferably via right femoral artery [15]. The angiograms and ventriculograms thus obtained were analyzed by an experienced hemodynamicist from our service, using a quantitative score system. After undergoing the procedure, these patients were divided into six subgroups: a) normal coronary arteries; b) narrow, tortuous coronary arteries; c) myocardial bridge or coronary spasm; d) coronary stenosis between 30–50%; e) coronary stenosis greater than 50%; and f) ejection fraction less than 50%. Patients with stenosis > 50% were considered with CAD.

### Statistical analysis

Continuous variables were reported as mean ± standard deviation (SD), and comparisons between groups were based on student t test. Categorical variables were summarized as percentage, and group comparisons were based on the Chi-square test. The logistic regression analyses were used with the aim of analyzing associations of the clinical variables (age, gender, systemic hypertension, dyslipidemia and diabetes mellitus), WM abnormalities and CI. The statistically significant data were considered an alpha error < 0.05. Statistical analyses were performed using the SPSS 13.0 (SPSS, Chicago, IL).

## Results

### Clinical characteristics

Of the 4042 patients who underwent EE, 1900 were men with a mean age of 56.5 ± 11.3 years. G1 group was constituted of 490 (12%) patients and G2 group of 3552 (88%). Dyslipidemia, systemic arterial hypertension, diabetes mellitus and familial history of CAD were more frequent (p < 0.001) in G1 group (Table 1).

**Table 1:** Comparison of the clinical findings in patients of the Group 1 (G1) and patients of the Group 2 (G2).

**VARIABLES**	**G1 n = 490 (12%)**	**G2 n = 3552 (88%)**	**P**
Age (Years)	60.1 ± 11.2	56 ± 11.2	< 0.0001
Gender (M/F)	222 (45%)/268 (55%)	1678 (47%)/1874 (53%)	0.421
BMI (Kg/m^2^)	28.2 ± 5.0	27.3 ± 4.5	0.914
Asymptomatic	101 (21%)	1191 (33%)	< 0.0001
Typical Angina	88 (18%)	261 (7%)	< 0.0001
Atypical Angina	301 (61%)	2100 (59%)	0.330
Systemic Hypertension	349 (71%)	1973 (55%)	< 0.0001
Dyslipidemia	376 (77%)	2468 (70%)	0.001
Cigarette Smoking	28 (6%)	181 (5%)	0.562
Diabetes Mellitus	79 (16%)	356 (10%)	< 0.0001
Family History of CAD	308 (63%)	1912 (54%)	< 0.0001
Previous Myocardial Infarction	43 (9%)	146 (4%)	< 0.0001
Coronary Bypass Surgery	61 (12%)	190 (5%)	< 0.0001
PCI	52 (11%)	191 (5%)	< 0.0001
Beta Blockers	167 (34%)	627 (18%)	< 0.0001
Calcium Channel Antagonists	45 (9%)	190 (5%)	0.001
Nitrates	49 (10%)	123 (3%)	< 0.0001
LBBB	17 (4%)	117 (3%)	0.833

G1 group: patients who failed to achieve 85% of the maximal age-predicted HR;

G2 group: patients who were able to achieve 85% of the maximal age-predicted HR.

BMI = Body Mass Index;

CAD = Coronary Arterial Disease

PCI = Percutaneous Coronary Intervention

LBBB = Left Bundle Branch Block

Between the groups there were not significant differences on atypical angina. However, regarding the asymptomatic patients, we observed greater frequency for G2 group. The typical angina was higher in frequency in G1 group. Coronary bypass surgery, percutaneous coronary intervention and carrying previous acute myocardial infarction were related to previous cardiovascular antecedents. All of them had a higher frequency in G1 group than in G2 group, with the exception of the LBBB, which did not have significant statistical difference. The use of beta-adrenergic blockers, calcium channel antagonists and nitrates were more frequent in G1 group than in G2 group (Table 1).

### Exercise echocardiography (EE)

The duration of the ET and the METS were less in the G1 group than in the G2 group. During maximal effort, it showed no significant difference of the systolic blood pressure. The wall motion score index (WMSI) was significantly higher in G1 group at rest and after the exercise (Table 2).

**Table 2:** Hemodynamic changes during the EE in patients: Group 1 (G1) and Group 2 (G2).

**VARIABLES**	**G1**	**G2**	**P**	**95% CI**
Resting Heart Rate	71.25 ± 13.1	77.6 ± 16.0	< 0.001	(-7.8 – -4.85)
Peak Heart Rate	118.0 ± 16.0	158.0 ± 18.4	< 0.0001	(-41.49 – -38.01)
Rest Systolic Arterial Pressure	130.0 ± 14.0	128.0 ± 32.0	0.183	(-9.58 – 5.01)
Peak Systolic Arterial Pressure	175.5 ± 24.8	189.1 ± 19.6	0.995	(-15.5 – -11.66)
Rest Diastolic Arterial Pressure	84.8 ± 38.6	82.9 ± 11.3	0.832	(0.28 – 3.55)
Peak Diastolic Arterial Pressure	87.0 ± 11.2	86.7 ± 9.6	0.484	(-0.05 – 1.32)
Ejection Fraction	0.64 ± 0.085	0.66 ± 0.065	< 0.0001	(-0.25 – -0.01)
WMSI, rest	1.06 ± 0.17	1.02 ± 0.09	< 0.0001	(0.03 – 0.05)
WMSI, after exercise	1.12 ± 0.23	1.04 ± 0.21	< 0.0001	(0.06 – 0.10)
Duration of the Test	4.8 ± 2.4	7.8 ± 2.8	< 0.0001	(-3.29 – -2.71)
METS	6.6 ± 2.2	9.3 ± 2.9	< 0.0001	(-1.67 – -1.88)

G1 group: patients who failed to achieve 85% of the maximal age-predicted HR;

G2 group: patients who were able to achieve 85% of the maximal age-predicted HR.

WMSI = Wall Motion Score Index

METS = Metabolic Equivalents

During the exercise, the presence of typical angina was more frequent in the G1 group. ST-segment depression had been observed more frequently in the G2 group. Severe arrhythmias (sustained ventricular tachycardia) occurred more frequently in the G1 group than in the G2 group. The arrhythmias (atrial and ventricular premature complexes) were similar in the 2 groups, as well as the necessity of interruption of the examination due to arterial hypertension (Table 3).

**Table 3:** Comparison of the clinical and electrocardiographic findings during Exercise Echocardiography in patients: Group 1 (G1) and Group 2 (G2).

**VARIABLES**	**G1 n = 490 (12%)**	**G2 n = 3552(88%)**	**P**
Angina	81 (16%)	150 (4%)	< 0.0001
Blood pressure ≥ 220 × 120 mmhg	72 (15%)	508 (14%)	0.822
Simple Arrhythmia	97 (20%)	822 (23%)	0.098
Severe Arrhythmia	20 (4%)	64 (2%)	0.001
ST segment changes	144 (30%)	1517 (43%)	< 0.0001

G1 group: patients who failed to achieve 85% of the maximal age-predicted HR;

G2 group: patients who were able to achieve 85% of the maximal age-predicted HR.

**Figure 1:**
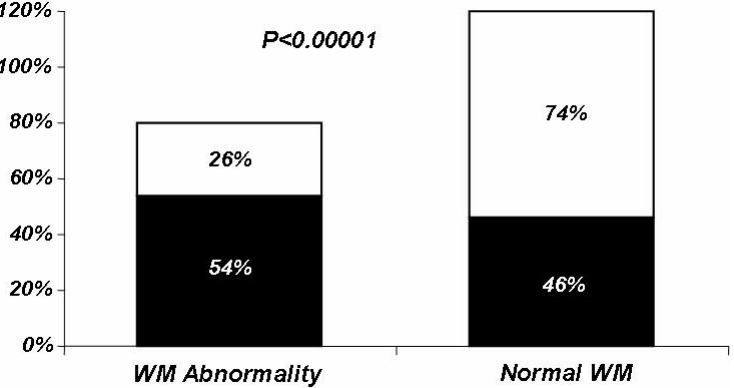
WM Abnormality in patients with and without chronotropic incompetence. G1 group (black): patients who failed to achieve 85% of the maximal age-predicted HR; G2 group (white): patients who were able to achieve 85% of the maximal age-predicted HR.

**Figure 2:**
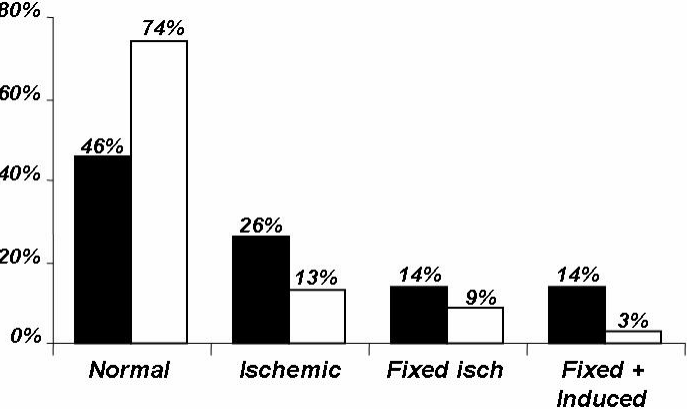
Exercise Echocardiography Results. G1 group (black): patients who failed to achieve 85% of the maximal age-predicted HR; G2 group (white): patients who were able to achieve 85% of the maximal age-predicted HR.

While the G2 group had more normal EE results, the G1 group had more ischemic results (Figure 1). The results of exercise echocardiography were demonstrated as normal, ischemic, fixed ischemic, and fixed and induced in the G1 and G2 groups (Figure 2).

### Coronary angiography (CA)

In G1 group, the CA was performed in 211 patients with positive EE for myocardial ischemia (80%), while in G2 group, the CA was performed in 644 patients (70%).

**Figure 3:**
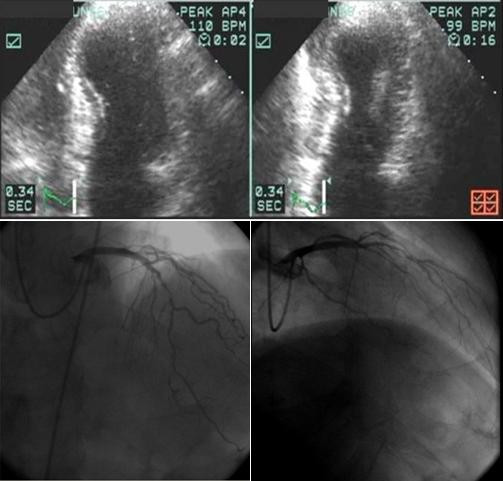
Patient, male, 57 years old with CI (85% of the maximal age-predicted HR = 139 beats per minute). He achieved HR = 110 beats per minute. In the EE, akinetic apical septum and hypokinetic antero-apical segment were observed and the CA demonstrated coronary stenosis greater than 50%.

According to the results (Table 4), it was observed that CAD [see additional file 1] was more frequent in G1 group than in G2 group (Figure 3).

**Table 4:** Comparison between the results of CA in patients with positive EE to myocardial ischemia: Group 1 (G1) versus Group 2 (G2).

**CA**	**G1 n = 490 (12%)**	**G2 n = 3552(88%)**	**P**	**Total**
Normal	14(6.6%)	72 (11.2%)	0.057	86
Coronary Crooked, Fine and with Parietals Injuries	02 (0.9%)	11 (1.7%)	0.43	13
Myocardium Bridges or Coronary Spasm	04 (1.9%)	11 (1.7%)	0.86	15
Coronary Stenosis 30–50%	12 (5.7%)	63 (9.8%)	0.068	75
Coronary Stenosis > 50%	173 (82%)	458 (71%)	0.0018	631
Ejection fraction < 50%	6 (2.8%)	28 (4.3%)	0.33	34

Total	211 (100%)	644 (100%)		855

G1 group: patients who failed to achieve 85% of the maximal age-predicted HR;

G2 group: patients who were able to achieve 85% of the maximal age-predicted HR.

The analyses of independent association of CAD with clinical characteristics (age, gender, systemic hypertension, dyslipidemia and diabetes mellitus), WM abnormality and CI were carried out by means of a logistic regression [see additional file 2]. It demonstrated significant elevated odds ration to WM abnormality, CI, gender, dyslipidemia, diabetes mellitus and age (Table 5).

**Table 5:** Logistic regression analyses of the clinical factors, WM abnormalities and CI contributing to CAD

**VARIABLES**	**OR Crude (IC 95%)**	**OR Adjusted IC 95%**	**P**
CI (G1)	3.69 (2.99 – 4.55)	2.62 (2.02 – 3.40)	< 0.00001
WM abnormality	16.61 (13.40 – 20.61)	13.55 (10.74 – 17.09)	< 0.00001
Gender (Male)	2.80 (2.34 – 3.35)	3.24 (2.62 – 4.01)	< 0.00001
Dyslipidemia	2.39 (1.92 – 2.99)	2.08 (1.60 – 2.69)	< 0.00001
Diabetes Mellitus	2.68 (2.14 – 3.36)	1.92 (1.46 – 2.54)	< 0.00001
Age	1.04 (1.03 – 1.04)	1.03 (1.02 – 1.04)	< 0.00001
Hypertension	1.61 (1.32 – 1.92)	* *	0.053

G1 group: patients who failed to achieve 85% of the maximal age-predicted HR;

CI = Chronotropic Incompetence

## Discussion

This study investigated, in a large cohort referred with known or suspected CAD, the hypothesis that there is an association between chronotropic incompetence and wall motion abnormality at rest and/or during exercise. The major findings of the present investigation were: (a) the frequency of WM abnormality evaluated by exercise echocardiography in patients with CI was marked higher than in patients who achieved at least 85% of the maximal age-predicted heart rate (54% versus 26%; P < 0.00001) (Figure 1); (b) CI was associated with a higher frequency of the 3 types of ischemic response (Figure 2) and (c) in patients with CI and with positive EE for myocardial ischemia, 82% had coronary stenosis greater than 50%, while in G2 group this value was only 71% (P = 0.0018) (Table 4).

Patients with CI had the higher frequency of cardiovascular risk factors such as diabetes mellitus, dyslipidemia and hypertension (Table 1), confirming the idea that CI has been associated with a higher cardiovascular risk [5]. The presence of typical angina and severe arrhythmias was more frequent in patients with chronotropic incompetence, demonstrating that CI is a spectrum of more severe CAD (Table 2, 3).

Nevertheless, ST segment depression was less frequent in patients with CI than in G2 group. Therefore, ST segment deviation is not good parameter to detect myocardial ischemia in CI patients. Moreover, this finding underlies that role of stress echocardiography in patients with CI. Furthermore, this investigation demonstrated that CI patients had three times more chance of having CAD (Table 5).

CI is a common finding observed during the accomplishment of treadmill exercise test (7 to 15%) [16,17]. Despite the suggestion that CI is an independent predictor of cardiovascular risk, it has not been used as a marker of cardiovascular risk yet [18]. The chronotropic response during the physical exercise reflects an extremely complex regulation which is correlated to age, functional capacity, heart rate under rest and autonomic balance [17]. Fukuma et al demonstrated that the baroreflex affects the heart rate response via an autonomic mechanism with depressed baroreflex sensibility [19]. In this research we found 490 (12%) patients with CI and we speculated if the baroreflex sensibility could be a possible mechanism to explain this result.

The heart rate recovery after exercise was considered another marker of cardiovascular risk. Reduced vagal activity has been shown to adversely impact mortality. Although attenuated heart rate recovery had not been helpful in predicting the presence of significant angiographic coronary disease, it predicted mortality [20]. In the present situation it was observed a decrease of the physiological increment of heart rate during exercise. It has been speculated vagal hyperactivity in order to decrease oxygen demand during exercise as a protective anti-ischemic mechanism [21]. This finding is in agreement with the data collected demonstrating that CI was helpful in predicting the presence of more severe coronary disease, although the CI impact in mortality has not been studied.

In addition, Ghaffari et al [22] observed the effect of intravenous atropine on treadmill stress test results on patients with CI. They concluded that the use of atropine as an adjunct to standard exercise stress test can help to decrease the number of non-concluded tests. But broader studies are necessary to define the role of atropine in exercise stress test and also to evaluate the accuracy of conclusive exercise stress test after atropine administration. Could be interesting speculate if exercise echocardiography wasn't better than atropine on treadmill stress to decreased the number of non-concluded tests.

Many researches had been showed that chronotropic incompetence during physical exercise was a more important predictor of cardiac death than myocardial ischemia evaluated by exercise test, stress echocardiography and PET Scan [1,3,17,18,23]. Although we have already had demonstrate that CI was helpful in predicting the presence of more severe coronary disease, we could to analyze the CI impact in mortality in the next investigate.

This finding of the present study extend our previous one that demonstrated that chronotropic incompetence in patients ≥ 65 years of age should not be underestimated or deemed physiological because it was associated with higher prevalence of WM abnormalities and, for instance, with myocardial ischemia [24]. Based on the data collected it is suggested that, even in younger patients with suspected CAD, chronotropic incompetence is a marker of more severe CAD.

A limitation of the present study was the fact that the frequency of beta blockers was more prevalent in CI patients. Although the drug had been discontinued four days earlier, its residual effect could not be excluded before the test.

## Conclusion

In summary, through the evaluation of patients for the exercise echocardiography, we suggest that: 1. Exercise echocardiography is a safe and very useful methodology in the evaluation of patients who fail to achieve 85% of the age-predicted HR; 2. The chronotropic incompetence, frequently observed during the exercise test, does not have to be underestimated or to be considered as physiological. The data collected suggest that CI is associated with higher frequency of wall motion abnormalities, reinforcing the concept that chronotropic incompetence is a marker of the severity of myocardial ischemia.

Although the CI mechanisms are not elucidated, this investigation suggests that chronotropic incompetence during treadmill should be used as a parameter of cardiovascular risk and not as an inconclusive find.

## Abbreviations

BMI = body mass index

CA = coronary angiography

CAD = coronary artery disease

CI = chronotropic incompetence

DVD = digital video display

ECG = electrocardiogram

EE = exercise echocardiography

ET = exercise test

HR = heart rate

LBBB = left bundle branch block

LV = left ventricular

METS = metabolic equivalents

PCI = percutaneous coronary intervention

WM = wall motion

WMSI = wall motion score index

## Competing interests

The author(s) declare that they have no competing interests.

## Authors' contributions

All authors contributed equally to this work, read and approved the final manuscript.

## Supplementary Material

Additional file 1 Image of CA demonstrated coronary stenosis greater than 50%. Example of a patient, male, 57 years old, with chronotropic incompetence. The CA demonstrated descendent anterior coronary stenosis greater than 50%. Click here for file

Additional file 2 Logistic regression analyses of the clinical factors, WM abnormalities and CI contributing to CAD – large tables. Data representing the statistical analysis of the factors associated with the diagnoses of CAD. Click here for file
