# Supplementary figures and images for: Chronotropic incompetence and a higher frequency of myocardial ischemia in exercise echocardiography
Source: Cardiovasc Ultrasound. 2007 Nov 2;5:38. doi: 10.1186/1476-7120-5-38 (PMC2186302; doi:10.1186/1476-7120-5-38)

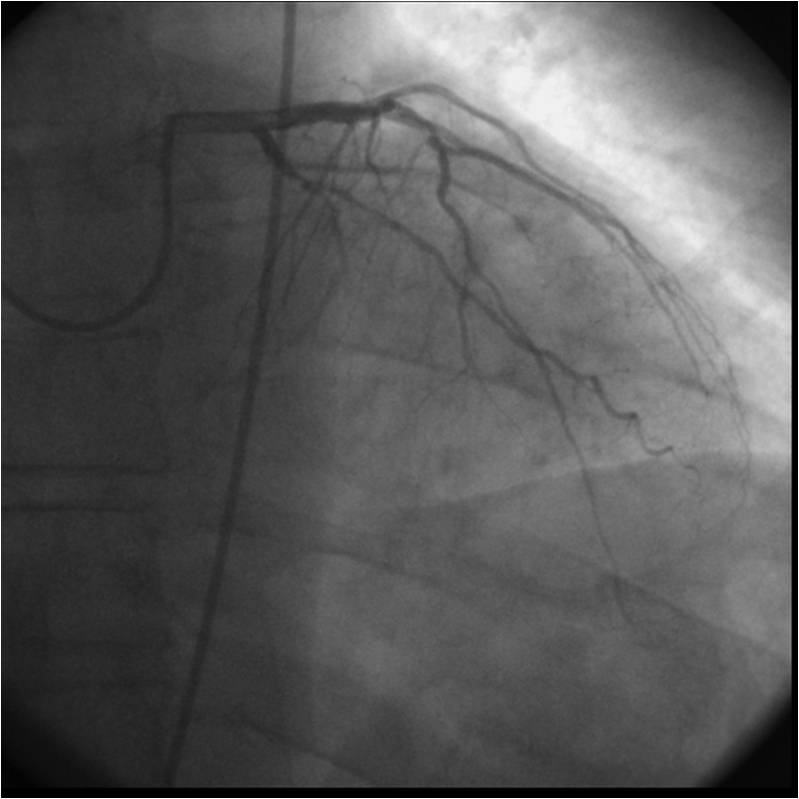

Supplement: Additional file 1 — Image of CA demonstrated coronary stenosis greater than 50%. Example of a patient, male, 57 years old, with chronotropic incompetence. The CA demonstrated descendent anterior coronary stenosis greater than 50%. [file 1476-7120-5-38-S1.jpeg]
