# Supplementary material for: Chronotropic incompetence and a higher frequency of myocardial ischemia in exercise echocardiography
Source: Cardiovasc Ultrasound. 2007 Nov 2;5:38. doi: 10.1186/1476-7120-5-38 (PMC2186302; doi:10.1186/1476-7120-5-38)
Supplement: Additional file 2 — Logistic regression analyses of the clinical factors, WM abnormalities and CI contributing to CAD – large tables. Data representing the statistical analysis of the factors associated with the diagnoses of CAD. [file 1476-7120-5-38-S2.doc]

**Logistic Regression**

**Notes**

| Output Created | | 28-JUL-2005 15:00:05 |
| --- | --- | --- |
| Comments | |  |
| Input | Data | C:\Documents and Settings\ENALDO\My Documents\CALCULOS ESTATISTICOS\TRAB JOSELINA E TAUANE\IC PICANO250707.sav |
|  | Filter | <none> |
|  | Weight | <none> |
|  | Split File | <none> |
|  | N of Rows in Working Data File | 4042 |
| Missing Value Handling | Definition of Missing | User-defined missing values are treated as missing |
| Syntax | | LOGISTIC REGRESSION CAT50  /METHOD = ENTER fmg  /CONTRAST (fmg)=Indicator  /PRINT = CORR CI(95)  /CRITERIA = PIN(.05) POUT(.10) ITERATE(20) CUT(.5) . |
| Resources | Elapsed Time | 0:00:00,23 |

**Case Processing Summary**

| Unweighted Cases(a) | | N | Percent |
| --- | --- | --- | --- |
| Selected Cases | Included in Analysis | 4042 | 100,0 |
|  | Missing Cases | 0 | ,0 |
|  | Total | 4042 | 100,0 |
| Unselected Cases | | 0 | ,0 |
| Total | | 4042 | 100,0 |

a If weight is in effect, see classification table for the total number of cases.

**Dependent Variable Encoding**

| Original Value | Internal Value |
| --- | --- |
| Coronary Stenosis <50% | 0 |
| Coronary Stenosis >50% | 1 |

**Categorical Variables Codings**

|  | | Frequency | Parameter coding |
| --- | --- | --- | --- |
|  |  |  | (1) |
| Heart Rate | G1 | 490 | 1,000 |
|  | G2 | 3552 | ,000 |

**Block 0: Beginning Block**

**Classification Table(a,b)**

|  | | | Observed | | | | Predicted |
| --- | --- | --- | --- | --- | --- | --- | --- |
|  |  |  | CAT50 | | Percentage Correct |  | |
|  |  |  | Coronary Stenosis <50% | Coronary Stenosis >50% |  |  | |
| Step 0 | CA50 | Coronary Stenosis <50% | 3411 | 0 | 100,0 |  | |
|  |  | Coronary Stenosis >50% | 631 | 0 | ,0 |  | |
|  | Overall Percentage | |  |  | 84,4 |  | |

a Constant is included in the model.

b The cut value is ,500

**Variables in the Equation**

|  | | B | S.E. | Wald | df | Sig. | Exp(B) |
| --- | --- | --- | --- | --- | --- | --- | --- |
|
| Step 0 | Constant | -1,687 | ,043 | 1516,279 | 1 | ,000 | ,185 |

**Variables not in the Equation**

|  | | | Score | df | Sig. |
| --- | --- | --- | --- | --- | --- |
| Step 0 | Variables | fmg(1) | 164,178 | 1 | ,000 |
|  | Overall Statistics | | 164,178 | 1 | ,000 |

**Block 1: Method = Enter**

**Omnibus Tests of Model Coefficients**

|  | |  | | Chi-square | | df | | Sig. |
| --- | --- | --- | --- | --- | --- | --- | --- | --- |
| Step 1 | Step | 134,803 | 1 | | ,000 | |  | |
|  | Block | 134,803 | 1 | | ,000 | |  | |
|  | Model | 134,803 | 1 | | ,000 | |  | |

**Model Summary**

| Step | -2 Log likelihood | Cox & Snell R Square | Nagelkerke R Square |
| --- | --- | --- | --- |
| 1 | 3366,895(a) | ,033 | ,057 |

a Estimation terminated at iteration number 5 because parameter estimates changed by less than ,001.

**Classification Table(a)**

|  | | | Observed | | | | Predicted |
| --- | --- | --- | --- | --- | --- | --- | --- |
|  |  |  | CAT50 | | Percentage Correct |  | |
|  |  |  | Coronary Stenosis <50% | Coronary Stenosis >50% |  |  | |
| Step 1 | CA50 | Coronary Stenosis <50% | 3411 | 0 | 100,0 |  | |
|  |  | Coronary Stenosis >50% | 631 | 0 | ,0 |  | |
|  | Overall Percentage | |  |  | 84,4 |  | |

a The cut value is ,500

**Variables in the Equation**

|  | | B | S.E. | Wald | df | Sig. | Exp(B) | 95,0% C.I.for EXP(B) | |
| --- | --- | --- | --- | --- | --- | --- | --- | --- | --- |
|  |  |  |  |  |  |  |  | Lower | Upper |
| Step 1(a) | fmg(1) | 1,305 | ,107 | 148,787 | 1 | .00000 | ***3.69*** | ***2.99*** | ***4.55*** |
|  | Constant | -1,910 | ,050 | 1455,925 | 1 | ,000 | ,148 |  |  |

a Variable(s) entered on step 1: fmg.

**Correlation Matrix**

|  | | Constant | fmg(1) |
| --- | --- | --- | --- |
| Step 1 | Constant | 1,000 | -,468 |
|  | fmg(1) | -,468 | 1,000 |

**Crosstabs**

**Notes**

| Output Created | | 28-JUL-2005 15:04:35 |
| --- | --- | --- |
| Comments | |  |
| Input | Data | C:\Documents and Settings\ENALDO\My Documents\CALCULOS ESTATISTICOS\TRAB JOSELINA E TAUANE\IC PICANO250707.sav |
|  | Filter | <none> |
|  | Weight | <none> |
|  | Split File | <none> |
|  | N of Rows in Working Data File | 4042 |
| Missing Value Handling | Definition of Missing | User-defined missing values are treated as missing. |
|  | Cases Used | Statistics for each table are based on all the cases with valid data in the specified range(s) for all variables in each table. |
| Syntax | | CROSSTABS  /TABLES=CAT50 BY efnormal  /FORMAT= AVALUE TABLES  /STATISTIC=CHISQ  /CELLS= COUNT COLUMN TOTAL  /COUNT ROUND CELL . |
| Resources | Elapsed Time | 0:00:00,11 |
|  | Dimensions Requested | 2 |
|  | Cells Available | 116508 |

**Case Processing Summary**

|  | Cases | | | | | |
| --- | --- | --- | --- | --- | --- | --- |
|  | Valid | | Missing | | Total | |
|  | N | Percent | N | Percent | N | Percent |
| CA50 * EE | 4042 | 100,0% | 0 | ,0% | 4042 | 100,0% |

**CAT50 * EEEF Crosstabulation**

|  | | | |  | | | | | | | EEEF | | Total |
| --- | --- | --- | --- | --- | --- | --- | --- | --- | --- | --- | --- | --- | --- |
|  |  |  |  |  |  |  |  |  |  |  | alterado | normal |  |
| CA50 | Coronary Stenosis <50% | Count | | 561 | | 2850 | | 3411 | |  | | | |
|  |  | % within EE | | 47,1% | | 100,0% | | 84,4% | |  | | | |
|  |  | % of Total | | 13,9% | | 70,5% | | 84,4% | |  | | | |
|  | Coronary Stenosis >50% | Count | | 631 | | 0 | | 631 | |  | | | |
|  |  | % within EE | | 52,9% | | ,0% | | 15,6% | |  | | | |
|  |  | % of Total | | 15,6% | | ,0% | | 15,6% | |  | | | |
| Total | | Count | | 1192 | | 2850 | | 4042 | |  | | | |
|  |  | % within EE | 100,0% | | 100,0% | | 100,0% | |  | | | | |
|  |  | % of Total | 29,5% | | 70,5% | | 100,0% | |  | | | | |

**Chi-Square Tests**

|  | Value | df | Asymp. Sig. (2-sided) | Exact Sig. (2-sided) | Exact Sig. (1-sided) |
| --- | --- | --- | --- | --- | --- |
| Pearson Chi-Square | 1787,774(b) | 1 | ,000 |  |  |
| Continuity Correction(a) | 1783,758 | 1 | ,000 |  |  |
| Likelihood Ratio | 1853,349 | 1 | ,000 |  |  |
| Fisher's Exact Test |  |  |  | ,000 | ,000 |
| Linear-by-Linear Association | 1787,331 | 1 | ,000 |  |  |
| N of Valid Cases | 4042 |  |  |  |  |

a Computed only for a 2x2 table

b 0 cells (,0%) have expected count less than 5. The minimum expected count is 186,08.

**Crosstabs**

**Notes**

| Output Created | | 28-JUL-2005 15:06:04 |
| --- | --- | --- |
| Comments | |  |
| Input | Data | C:\Documents and Settings\ENALDO\My Documents\CALCULOS ESTATISTICOS\TRAB JOSELINA E TAUANE\IC PICANO250707.sav |
|  | Filter | <none> |
|  | Weight | <none> |
|  | Split File | <none> |
|  | N of Rows in Working Data File | 4042 |
| Missing Value Handling | Definition of Missing | User-defined missing values are treated as missing. |
|  | Cases Used | Statistics for each table are based on all the cases with valid data in the specified range(s) for all variables in each table. |
| Syntax | | CROSSTABS  /TABLES=CAT50 BY altesfor  /FORMAT= AVALUE TABLES  /STATISTIC=CHISQ  /CELLS= COUNT COLUMN TOTAL  /COUNT ROUND CELL . |
| Resources | Elapsed Time | 0:00:00,11 |
|  | Dimensions Requested | 2 |
|  | Cells Available | 116508 |

**Case Processing Summary**

|  | Cases | | | | | |
| --- | --- | --- | --- | --- | --- | --- |
|  | Valid | | Missing | | Total | |
|  | N | Percent | N | Percent | N | Percent |
| CA50 WM abnormality | 4042 | 100,0% | 0 | ,0% | 4042 | 100,0% |

**CAT50 WM abnormality * Crosstabulation**

|  | | | | WM abnormality | | | Total | |  |
| --- | --- | --- | --- | --- | --- | --- | --- | --- | --- |
|  | | | | falso | verdadeiro | |  | |  |
| CA50 | Coronary Stenosis <50% | Count | | 3227 | 184 | | 3411 | |  |
|  |  | % within WM abnormality | | 90,9% | 37,5% | | 84,4% | |  |
|  |  | % of Total | | 79,8% | 4,6% | | 84,4% | |  |
|  | Coronary Stenosis >50% | Count | | 324 | 307 | | 631 | |  |
|  |  | % within WM abnormality | | 9,1% | 62,5% | | 15,6% | |  |
|  |  | % of Total | | 8,0% | 7,6% | | 15,6% | |  |
| Total | | Count | | 3551 | 491 | | 4042 | |  |
|  | % within WM abnormality | | 100,0% | | | 100,0% | | 100,0% | |
|  | % of Total | | 87,9% | | | 12,1% | | 100,0% | |

**Chi-Square Tests**

|  | Value | df | Asymp. Sig. (2-sided) | Exact Sig. (2-sided) | Exact Sig. (1-sided) |
| --- | --- | --- | --- | --- | --- |
| Pearson Chi-Square | 933,728(b) | 1 | ,000 |  |  |
| Continuity Correction(a) | 929,679 | 1 | ,000 |  |  |
| Likelihood Ratio | 683,208 | 1 | ,000 |  |  |
| Fisher's Exact Test |  |  |  | ,000 | ,000 |
| Linear-by-Linear Association | 933,497 | 1 | ,000 |  |  |
| N of Valid Cases | 4042 |  |  |  |  |

a Computed only for a 2x2 table

b 0 cells (,0%) have expected count less than 5. The minimum expected count is 76,65.

**Logistic Regression**

**Notes**

| Output Created | | 28-JUL-2005 15:07:06 |
| --- | --- | --- |
| Comments | |  |
| Input | Data | C:\Documents and Settings\ENALDO\My Documents\CALCULOS ESTATISTICOS\TRAB JOSELINA E TAUANE\IC PICANO250707.sav |
|  | Filter | <none> |
|  | Weight | <none> |
|  | Split File | <none> |
|  | N of Rows in Working Data File | 4042 |
| Missing Value Handling | Definition of Missing | User-defined missing values are treated as missing |
| Syntax | | LOGISTIC REGRESSION CAT50  /METHOD = ENTER altesfor  /CONTRAST (altesfor)=Indicator(1)  /PRINT = CORR CI(95)  /CRITERIA = PIN(.05) POUT(.10) ITERATE(20) CUT(.5) . |
| Resources | Elapsed Time | 0:00:00,23 |

**Case Processing Summary**

| Unweighted Cases(a) | | N | Percent |
| --- | --- | --- | --- |
| Selected Cases | Included in Analysis | 4042 | 100,0 |
|  | Missing Cases | 0 | ,0 |
|  | Total | 4042 | 100,0 |
| Unselected Cases | | 0 | ,0 |
| Total | | 4042 | 100,0 |

a If weight is in effect, see classification table for the total number of cases.

**Dependent Variable Encoding**

| Original Value | Internal Value |
| --- | --- |
| Coronary Stenosis <50% | 0 |
| Coronary Stenosis >50% | 1 |

**Categorical Variables Codings**

|  | | Frequency | Parameter coding |
| --- | --- | --- | --- |
|  |  |  | (1) |
| WM abnormality | falso | 3551 | ,000 |
|  | verdadeiro | 491 | 1,000 |

**Block 0: Beginning Block**

**Classification Table(a,b)**

|  | | | Observed | | | | Predicted |
| --- | --- | --- | --- | --- | --- | --- | --- |
|  |  |  | CA50 | | Percentage Correct |  | |
|  |  |  | Coronary Stenosis <50% | Coronary Stenosis >50% |  |  | |
| Step 0 | CA50 | Coronary Stenosis <50% | 3411 | 0 | 100,0 |  | |
|  |  | Coronary Stenosis >50% | 631 | 0 | ,0 |  | |
|  | Overall Percentage | |  |  | 84,4 |  | |

a Constant is included in the model.

b The cut value is ,500

**Variables in the Equation**

|  | | B | S.E. | Wald | df | Sig. | Exp(B) |
| --- | --- | --- | --- | --- | --- | --- | --- |
|
| Step 0 | Constant | -1,687 | ,043 | 1516,279 | 1 | ,000 | ,185 |

**Variables not in the Equation**

|  | | | Score | df | Sig. |
| --- | --- | --- | --- | --- | --- |
| Step 0 | Variables | altesfor(1) | 933,728 | 1 | ,000 |
|  | Overall Statistics | | 933,728 | 1 | ,000 |

**Block 1: Method = Enter**

**Omnibus Tests of Model Coefficients**

|  | |  | | Chi-square | | df | | Sig. |
| --- | --- | --- | --- | --- | --- | --- | --- | --- |
| Step 1 | Step | 683,208 | 1 | | ,000 | |  | |
|  | Block | 683,208 | 1 | | ,000 | |  | |
|  | Model | 683,208 | 1 | | ,000 | |  | |

**Model Summary**

| Step | -2 Log likelihood | Cox & Snell R Square | Nagelkerke R Square |
| --- | --- | --- | --- |
| 1 | 2818,491(a) | ,156 | ,268 |

a Estimation terminated at iteration number 5 because parameter estimates changed by less than ,001.

**Classification Table(a)**

|  | | | Observed | | | | Predicted |
| --- | --- | --- | --- | --- | --- | --- | --- |
|  |  |  | CA50 | | Percentage Correct |  | |
|  |  |  | Coronary Stenosis<50% | Coronary Stenosis >50% |  |  | |
| Step 1 | CA50 | Coronary Stenosis <50% | 3227 | 184 | 94,6 |  | |
|  |  | Coronary Stenosis >50% | 324 | 307 | 48,7 |  | |
|  | Overall Percentage | |  |  | 87,4 |  | |

a The cut value is ,500

**Variables in the Equation**

|  | | B | S.E. | Wald | df | Sig. | Exp(B) | 95,0% C.I.for EXP(B) | |
| --- | --- | --- | --- | --- | --- | --- | --- | --- | --- |
|  |  |  |  |  |  |  |  | Lower | Upper |
| Step 1(a) | WM abnormality (1) | 2,810 | ,110 | 653,417 | 1 | ***.000000*** | ***16.62*** | ***13.40*** | ***20.61*** |
|  | Constant | -2,299 | ,058 | 1555,632 | 1 | ,000 | ,100 |  |  |

a Variable(s) entered on step 1: altesfor.

**Correlation Matrix**

|  | | Constant | altesfor(1) |
| --- | --- | --- | --- |
| Step 1 | Constant | 1,000 | -,530 |
|  | WM abnormality (1) | -,530 | 1,000 |

**Logistic Regression**

**Notes**

| Output Created | | 28-JUL-2005 15:42:34 |
| --- | --- | --- |
| Comments | |  |
| Input | Data | C:\Documents and Settings\ENALDO\My Documents\CALCULOS ESTATISTICOS\TRAB JOSELINA E TAUANE\IC PICANO250707.sav |
|  | Filter | <none> |
|  | Weight | <none> |
|  | Split File | <none> |
|  | N of Rows in Working Data File | 4042 |
| Missing Value Handling | Definition of Missing | User-defined missing values are treated as missing |
| Syntax | | LOGISTIC REGRESSION CAT50  /METHOD = ENTER altesfor fmg sexo idade imc hiperten diabetes dislipid tabagism  /CONTRAST (altesfor)=Indicator(1) /CONTRAST (fmg)=Indicator /CONTRAST (sexo)=Indicator(1) /CONTRAST (hiperten)=Indicator  (1) /CONTRAST (diabetes)=Indicator(1) /CONTRAST (dislipid)=Indicator(1) /CONTRAST (tabagism)=Indicator(1)  /PRINT = CORR CI(95)  /CRITERIA = PIN(.05) POUT(.10) ITERATE(20) CUT(.5) . |
| Resources | Elapsed Time | 0:00:00,31 |

**Case Processing Summary**

| Unweighted Cases(a) | | N | Percent |
| --- | --- | --- | --- |
| Selected Cases | Included in Analysis | 3941 | 97,5 |
|  | Missing Cases | 101 | 2,5 |
|  | Total | 4042 | 100,0 |
| Unselected Cases | | 0 | ,0 |
| Total | | 4042 | 100,0 |

a If weight is in effect, see classification table for the total number of cases.

**Dependent Variable Encoding**

| Original Value | Internal Value |
| --- | --- |
| Coronary Stenosis <50% | 0 |
| Coronary Stenosis >50% | 1 |

**Categorical Variables Codings**

|  | | Frequency | Parameter coding |
| --- | --- | --- | --- |
|  |  |  | (1) |
| Cigarrete smoking | falso | 3743 | ,000 |
|  | verdadeiro | 198 | 1,000 |
| Heart Hate | G1 | 477 | 1,000 |
|  | G2 | 3464 | ,000 |
| sexo | F | 2082 | ,000 |
|  | M | 1859 | 1,000 |
| HYPERTENSION | falso | 1671 | ,000 |
|  | verdadeiro | 2270 | 1,000 |
| DIABETES MELLITUS | falso | 3522 | ,000 |
|  | verdadeiro | 419 | 1,000 |
| DYSLIPIDEMIA | falso | 1168 | ,000 |
|  | verdadeiro | 2773 | 1,000 |
| WM abnormality | falso | 3470 | ,000 |
|  | verdadeiro | 471 | 1,000 |

**Block 0: Beginning Block**

**Classification Table(a,b)**

|  | | | Observed | | | | Predicted |
| --- | --- | --- | --- | --- | --- | --- | --- |
|  |  |  | CA50 | | Percentage Correct |  | |
|  |  |  | Coronary Stenosis<50% | Coronary Stenosis >50% |  |  | |
| Step 0 | CA50 | Coronary Stenosis <50% | 3324 | 0 | 100,0 |  | |
|  |  | Coronary Stenosis >50% | 617 | 0 | ,0 |  | |
|  | Overall Percentage | |  |  | 84,3 |  | |

a Constant is included in the model.

b The cut value is ,500

**Variables in the Equation**

|  | | B | S.E. | Wald | df | Sig. | Exp(B) |
| --- | --- | --- | --- | --- | --- | --- | --- |
|
| Step 0 | Constant | -1,684 | ,044 | 1475,884 | 1 | ,000 | ,186 |

**Variables not in the Equation**

|  | | | Score | df | Sig. |
| --- | --- | --- | --- | --- | --- |
| Step 0 | Variables | WM abnormality (1) | 910,221 | 1 | ,000 |
|  |  | fmg(1) | 164,118 | 1 | ,000 |
|  |  | sex (1) | 130,229 | 1 | ,000 |
|  |  | age | 84,565 | 1 | ,000 |
|  |  | BMI | 4,487 | 1 | ,034 |
|  |  | hyperten(1) | 27,959 | 1 | ,000 |
|  |  | diabetes(1) | 81,296 | 1 | ,000 |
|  |  | dyslipid(1) | 61,750 | 1 | ,000 |
|  |  | tabagism(1) | 9,062 | 1 | ,003 |
|  | Overall Statistics | | 1141,478 | 9 | ,000 |

**Block 1: Method = Enter**

**Omnibus Tests of Model Coefficients**

|  | |  | | Chi-square | | df | | Sig. |
| --- | --- | --- | --- | --- | --- | --- | --- | --- |
| Step 1 | Step | 959,784 | 9 | | ,000 | |  | |
|  | Block | 959,784 | 9 | | ,000 | |  | |
|  | Model | 959,784 | 9 | | ,000 | |  | |

**Model Summary**

| Step | -2 Log likelihood | Cox & Snell R Square | Nagelkerke R Square |
| --- | --- | --- | --- |
| 1 | 2460,374(a) | ,216 | ,373 |

a Estimation terminated at iteration number 6 because parameter estimates changed by less than ,001.

**Classification Table(a)**

|  | | | Observed | | | | Predicted |
| --- | --- | --- | --- | --- | --- | --- | --- |
|  |  |  | CA50 | | Percentage Correct |  | |
|  |  |  | Coronary Stenosis<50% | Coronary Stenosis >50% |  |  | |
| Step 1 | CA50 | Coronary Stenosis <50% | 3228 | 96 | 97,1 |  | |
|  |  | Coronary Stenosis >50% | 366 | 251 | 40,7 |  | |
|  | Overall Percentage | |  |  | 88,3 |  | |

a The cut value is ,500

**Variables in the Equation**

|  | | B | S.E. | Wald | df | Sig. | Exp(B) | 95,0% C.I.for EXP(B) | |
| --- | --- | --- | --- | --- | --- | --- | --- | --- | --- |
|  |  |  |  |  |  |  |  | Lower | Upper |
| Step 1(a) | WM abnormality (1) | 2,617 | ,122 | 459,622 | 1 | ,000 | ***13.69*** | ***10.78*** | ***17.39*** |
|  | fmg(1) | ,998 | ,135 | 54,526 | 1 | ,000 | ***2.71*** | ***2.08*** | ***3.54*** |
|  | sex(1) | 1,182 | ,111 | 113,059 | 1 | ,000 | ***3.26*** | ***2.62*** | ***4.05*** |
|  | age | ,027 | ,005 | 30,528 | 1 | ,000 | ***1.03*** | ***1.02*** | ***1.04*** |
|  | BMI | -,044 | ,012 | 12,570 | 1 | ,000 | ***.96*** | ***.93*** | ***.98*** |
|  | hyperten(1) | ,298 | ,115 | 6,673 | 1 | ,010 | ***1.35*** | ***1.07*** | ***1.69*** |
|  | diabetes(1) | ,664 | ,145 | 21,108 | 1 | ,000 | ***1.94*** | ***1.46*** | ***2.58*** |
|  | dyslipid(1) | ,736 | ,136 | 29,437 | 1 | ,000 | ***2.09*** | ***1.60*** | ***2.72*** |
|  | tabagism(1) | ,234 | ,220 | 1,139 | 1 | ,286 | ***1.26*** | ***.82*** | ***1.94*** |
|  | Constant | -4,322 | ,474 | 83,245 | 1 | ,000 | ,013 |  |  |

a Variable(s) entered on step 1: altesfor, fmg, sexo, idade, imc, hiperten, diabetes, dislipid, tabagism.

**Correlation Matrix**

|  | | Constant | altesfor(1) | fmg(1) | sexo(1) | idade | imc | hiperten(1) | diabetes(1) | dislipid(1) | tabagism(1) |
| --- | --- | --- | --- | --- | --- | --- | --- | --- | --- | --- | --- |
| Step 1 | Constant | 1,000 | -,049 | ,048 | -,234 | -,683 | -,712 | ,068 | ,054 | -,161 | -,087 |
|  | WM abnormality (1) | -,049 | 1,000 | -,020 | ,122 | -,019 | -,050 | ,076 | -,008 | ,015 | -,057 |
|  | fmg(1) | ,048 | -,020 | 1,000 | ,071 | -,081 | -,077 | -,074 | ,002 | ,016 | ,003 |
|  | sex(1) | -,234 | ,122 | ,071 | 1,000 | ,120 | -,014 | ,019 | ,003 | ,039 | -,054 |
|  | age | -,683 | -,019 | -,081 | ,120 | 1,000 | ,104 | -,117 | -,068 | -,007 | ,094 |
|  | BMI | -,712 | -,050 | -,077 | -,014 | ,104 | 1,000 | -,178 | -,072 | -,081 | ,006 |
|  | hyperten(1) | ,068 | ,076 | -,074 | ,019 | -,117 | -,178 | 1,000 | -,083 | -,121 | -,002 |
|  | diabetes(1) | ,054 | -,008 | ,002 | ,003 | -,068 | -,072 | -,083 | 1,000 | ,015 | -,035 |
|  | dyslipid(1) | -,161 | ,015 | ,016 | ,039 | -,007 | -,081 | -,121 | ,015 | 1,000 | ,042 |
|  | Cigarrete smoking(1) | -,087 | -,057 | ,003 | -,054 | ,094 | ,006 | -,002 | -,035 | ,042 | 1,000 |

**Logistic Regression**

**Notes**

| Output Created | | 09-AUG-2005 22:03:23 |
| --- | --- | --- |
| Comments | |  |
| Input | Data | C:\Documents and Settings\ENALDO\My Documents\CALCULOS ESTATISTICOS\TRAB JOSELINA E TAUANE\IC PICANO 280707modifi.sav |
|  | Filter | <none> |
|  | Weight | <none> |
|  | Split File | <none> |
|  | N of Rows in Working Data File | 4042 |
| Missing Value Handling | Definition of Missing | User-defined missing values are treated as missing |
| Syntax | | LOGISTIC REGRESSION CAT50  /METHOD = ENTER altesfor  /CONTRAST (altesfor)=Indicator(1)  /PRINT = CORR CI(95)  /CRITERIA = PIN(.05) POUT(.10) ITERATE(20) CUT(.5) . |
| Resources | Elapsed Time | 0:00:00.17 |

**Case Processing Summary**

| Unweighted Cases(a) | | N | Percent |
| --- | --- | --- | --- |
| Selected Cases | Included in Analysis | 4042 | 100.0 |
|  | Missing Cases | 0 | .0 |
|  | Total | 4042 | 100.0 |
| Unselected Cases | | 0 | .0 |
| Total | | 4042 | 100.0 |

a If weight is in effect, see classification table for the total number of cases.

**Dependent Variable Encoding**

| Original Value | Internal Value |
| --- | --- |
| Coronary Stenosis <50% | 0 |
| Coronary Stenosis >50% | 1 |

**Categorical Variables Codings**

|  | | Frequency | Parameter coding |
| --- | --- | --- | --- |
|  |  |  | (1) |
| WM abnormality | falso | 3551 | .000 |
|  | verdadeiro | 491 | 1.000 |

**Block 0: Beginning Block**

**Classification Table(a,b)**

|  | | | Observed | | | | Predicted |
| --- | --- | --- | --- | --- | --- | --- | --- |
|  |  |  | CA50 | | Percentage Correct |  | |
|  |  |  | Coronary Stenosis<50% | Coronary Stenosis >50% |  |  | |
| Step 0 | CA50 | Coronary Stenosis<50% | 3411 | 0 | 100.0 |  | |
|  |  | Coronary Stenosis >50% | 631 | 0 | .0 |  | |
|  | Overall Percentage | |  |  | 84.4 |  | |

a Constant is included in the model.

b The cut value is .500

**Variables in the Equation**

|  | | B | S.E. | Wald | df | Sig. | Exp(B) |
| --- | --- | --- | --- | --- | --- | --- | --- |
|
| Step 0 | Constant | -1.687 | .043 | 1516.279 | 1 | .000 | .185 |

**Variables not in the Equation**

|  | | | Score | df | Sig. |
| --- | --- | --- | --- | --- | --- |
| Step 0 | Variables | WM abnormality (1) | 933.728 | 1 | .000 |
|  | Overall Statistics | | 933.728 | 1 | .000 |

**Block 1: Method = Enter**

**Omnibus Tests of Model Coefficients**

|  | |  | | Chi-square | | df | | Sig. |
| --- | --- | --- | --- | --- | --- | --- | --- | --- |
| Step 1 | Step | 683.208 | 1 | | .000 | |  | |
|  | Block | 683.208 | 1 | | .000 | |  | |
|  | Model | 683.208 | 1 | | .000 | |  | |

**Model Summary**

| Step | -2 Log likelihood | Cox & Snell R Square | Nagelkerke R Square |
| --- | --- | --- | --- |
| 1 | 2818.491(a) | .156 | .268 |

a Estimation terminated at iteration number 5 because parameter estimates changed by less than .001.

**Classification Table(a)**

|  | | | Observed | | | | Predicted |
| --- | --- | --- | --- | --- | --- | --- | --- |
|  |  |  | CAT50 | | Percentage Correct |  | |
|  |  |  | Coronary Stenosis<50% | Coronary Stenosis >50% |  |  | |
| Step 1 | CA50 | Coronary Stenosis <50% | 3227 | 184 | 94.6 |  | |
|  |  | Coronary Stenosis >50% | 324 | 307 | 48.7 |  | |
|  | Overall Percentage | |  |  | 87.4 |  | |

a The cut value is .500

**Variables in the Equation**

|  | | B | S.E. | Wald | df | Sig. | Exp(B) | 95.0% C.I.for EXP(B) | |
| --- | --- | --- | --- | --- | --- | --- | --- | --- | --- |
|  |  |  |  |  |  |  |  | Lower | Upper |
| Step 1(a) | altesfor(1) | 2.810 | .110 | 653.417 | 1 | ***.000000*** | ***16.62*** | ***13.40*** | ***20.61*** |
|  | Constant | -2.299 | .058 | 1555.632 | 1 | .000 | .100 |  |  |

a Variable(s) entered on step 1: altesfor.

**Correlation Matrix**

|  | | Constant | altesfor(1) |
| --- | --- | --- | --- |
| Step 1 | Constant | 1.000 | -.530 |
|  | WM abnormality (1) | -.530 | 1.000 |

**Logistic Regression**

**Notes**

| Output Created | | 09-AUG-2005 22:05:17 |
| --- | --- | --- |
| Comments | |  |
| Input | Data | C:\Documents and Settings\ENALDO\My Documents\CALCULOS ESTATISTICOS\TRAB JOSELINA E TAUANE\IC PICANO 280707modifi.sav |
|  | Filter | <none> |
|  | Weight | <none> |
|  | Split File | <none> |
|  | N of Rows in Working Data File | 4042 |
| Missing Value Handling | Definition of Missing | User-defined missing values are treated as missing |
| Syntax | | LOGISTIC REGRESSION CAT50  /METHOD = ENTER fmg  /CONTRAST (fmg)=Indicator  /PRINT = CORR CI(95)  /CRITERIA = PIN(.05) POUT(.10) ITERATE(20) CUT(.5) . |
| Resources | Elapsed Time | 0:00:00.16 |

**Case Processing Summary**

| Unweighted Cases(a) | | N | Percent |
| --- | --- | --- | --- |
| Selected Cases | Included in Analysis | 4042 | 100.0 |
|  | Missing Cases | 0 | .0 |
|  | Total | 4042 | 100.0 |
| Unselected Cases | | 0 | .0 |
| Total | | 4042 | 100.0 |

a If weight is in effect, see classification table for the total number of cases.

**Dependent Variable Encoding**

| Original Value | Internal Value |
| --- | --- |
| Coronary Stenosis <50% | 0 |
| Coronary Stenosis >50% | 1 |

**Categorical Variables Codings**

|  | | Frequency | Parameter coding |
| --- | --- | --- | --- |
|  |  |  | (1) |
| Heart hate | G1 | 490 | 1.000 |
|  | G2 | 3552 | .000 |

**Block 0: Beginning Block**

**Classification Table(a,b)**

|  | | | Observed | | | | Predicted |
| --- | --- | --- | --- | --- | --- | --- | --- |
|  |  |  | CAT50 | | Percentage Correct |  | |
|  |  |  | Coronary Stenosis <50% | Coronary Stenosis >50% |  |  | |
| Step 0 | CA50 | Coronary Stenosis <50% | 3411 | 0 | 100.0 |  | |
|  |  | Coronary Stenosis >50% | 631 | 0 | .0 |  | |
|  | Overall Percentage | |  |  | 84.4 |  | |

a Constant is included in the model.

b The cut value is .500

**Variables in the Equation**

|  | | B | S.E. | Wald | df | Sig. | Exp(B) |
| --- | --- | --- | --- | --- | --- | --- | --- |
|
| Step 0 | Constant | -1.687 | .043 | 1516.279 | 1 | .000 | .185 |

**Variables not in the Equation**

|  | | | Score | df | Sig. |
| --- | --- | --- | --- | --- | --- |
| Step 0 | Variables | fmg(1) | 164.178 | 1 | .000 |
|  | Overall Statistics | | 164.178 | 1 | .000 |

**Block 1: Method = Enter**

**Omnibus Tests of Model Coefficients**

|  | |  | | Chi-square | | df | | Sig. |
| --- | --- | --- | --- | --- | --- | --- | --- | --- |
| Step 1 | Step | 134.803 | 1 | | .000 | |  | |
|  | Block | 134.803 | 1 | | .000 | |  | |
|  | Model | 134.803 | 1 | | .000 | |  | |

**Model Summary**

| Step | -2 Log likelihood | Cox & Snell R Square | Nagelkerke R Square |
| --- | --- | --- | --- |
| 1 | 3366.895(a) | .033 | .057 |

a Estimation terminated at iteration number 5 because parameter estimates changed by less than .001.

**Classification Table(a)**

|  | | | Observed | | | | Predicted |
| --- | --- | --- | --- | --- | --- | --- | --- |
|  |  |  | CAT50 | | Percentage Correct |  | |
|  |  |  | Coronary Stenosis <50% | Coronary Stenosis >50% |  |  | |
| Step 1 | CA50 | Coronary Stenosis <50% | 3411 | 0 | 100.0 |  | |
|  |  | Coronary Stenosis >50% | 631 | 0 | .0 |  | |
|  | Overall Percentage | |  |  | 84.4 |  | |

a The cut value is .500

**Variables in the Equation**

|  | | B | S.E. | Wald | df | Sig. | Exp(B) | 95.0% C.I.for EXP(B) | |
| --- | --- | --- | --- | --- | --- | --- | --- | --- | --- |
|  |  |  |  |  |  |  |  | Lower | Upper |
| Step 1(a) | fmg(1) | 1.305 | .107 | 148.787 | 1 | ***.000000*** | ***3.69*** | ***2.99*** | ***4.55*** |
|  | Constant | -1.910 | .050 | 1455.925 | 1 | .000 | .148 |  |  |

a Variable(s) entered on step 1: fmg.

**Correlation Matrix**

|  | | Constant | fmg(1) |
| --- | --- | --- | --- |
| Step 1 | Constant | 1.000 | -.468 |
|  | fmg(1) | -.468 | 1.000 |

**Logistic Regression**

**Notes**

| Output Created | | 09-AUG-2005 22:07:16 |
| --- | --- | --- |
| Comments | |  |
| Input | Data | C:\Documents and Settings\ENALDO\My Documents\CALCULOS ESTATISTICOS\TRAB JOSELINA E TAUANE\IC PICANO 280707modifi.sav |
|  | Filter | <none> |
|  | Weight | <none> |
|  | Split File | <none> |
|  | N of Rows in Working Data File | 4042 |
| Missing Value Handling | Definition of Missing | User-defined missing values are treated as missing |
| Syntax | | LOGISTIC REGRESSION CAT50  /METHOD = ENTER sexo  /CONTRAST (sexo)=Indicator(1)  /PRINT = CORR CI(95)  /CRITERIA = PIN(.05) POUT(.10) ITERATE(20) CUT(.5) . |
| Resources | Elapsed Time | 0:00:00.16 |

**Case Processing Summary**

| Unweighted Cases(a) | | N | Percent |
| --- | --- | --- | --- |
| Selected Cases | Included in Analysis | 4042 | 100.0 |
|  | Missing Cases | 0 | .0 |
|  | Total | 4042 | 100.0 |
| Unselected Cases | | 0 | .0 |
| Total | | 4042 | 100.0 |

a If weight is in effect, see classification table for the total number of cases.

**Dependent Variable Encoding**

| Original Value | Internal Value |
| --- | --- |
| Coronary Stenosis 30–50% | 0 |
| LESÃO MAIOR DE 50% | 1 |

**Categorical Variables Codings**

|  | | Frequency | Parameter coding |
| --- | --- | --- | --- |
|  |  |  | (1) |
| sexo | F | 2142 | .000 |
|  | M | 1900 | 1.000 |

**Block 0: Beginning Block**

**Classification Table(a,b)**

|  | | | Observed | | | | Predicted |
| --- | --- | --- | --- | --- | --- | --- | --- |
|  |  |  | CA50 | | Percentage Correct |  | |
|  |  |  | Coronary Stenosis <50% | Coronary Stenosis >50% |  |  | |
| Step 0 | CA50 | Coronary Stenosis <50% | 3411 | 0 | 100.0 |  | |
|  |  | Coronary Stenosis >50% | 631 | 0 | .0 |  | |
|  | Overall Percentage | |  |  | 84.4 |  | |

a Constant is included in the model.

b The cut value is .500

**Variables in the Equation**

|  | | B | S.E. | Wald | df | Sig. | Exp(B) |
| --- | --- | --- | --- | --- | --- | --- | --- |
|
| Step 0 | Constant | -1.687 | .043 | 1516.279 | 1 | .000 | .185 |

**Variables not in the Equation**

|  | | | Score | df | Sig. |
| --- | --- | --- | --- | --- | --- |
| Step 0 | Variables | sexo(1) | 132.133 | 1 | .000 |
|  | Overall Statistics | | 132.133 | 1 | .000 |

**Block 1: Method = Enter**

**Omnibus Tests of Model Coefficients**

|  | |  | | Chi-square | | df | | Sig. |
| --- | --- | --- | --- | --- | --- | --- | --- | --- |
| Step 1 | Step | 133.715 | 1 | | .000 | |  | |
|  | Block | 133.715 | 1 | | .000 | |  | |
|  | Model | 133.715 | 1 | | .000 | |  | |

**Model Summary**

| Step | -2 Log likelihood | Cox & Snell R Square | Nagelkerke R Square |
| --- | --- | --- | --- |
| 1 | 3367.984(a) | .033 | .056 |

a Estimation terminated at iteration number 5 because parameter estimates changed by less than .001.

**Classification Table(a)**

|  | | | Observed | | | | Predicted |
| --- | --- | --- | --- | --- | --- | --- | --- |
|  |  |  | CA50 | | Percentage Correct |  | |
|  |  |  | Coronary Stenosis <50% | Coronary Stenosis >50% |  |  | |
| Step 1 | CA50 | Coronary Stenosis <50% | 3411 | 0 | 100.0 |  | |
|  |  | Coronary Stenosis >50% | 631 | 0 | .0 |  | |
|  | Overall Percentage | |  |  | 84.4 |  | |

a The cut value is .500

**Variables in the Equation**

|  | | B | S.E. | Wald | df | Sig. | Exp(B) | 95.0% C.I.for EXP(B) | |
| --- | --- | --- | --- | --- | --- | --- | --- | --- | --- |
|  |  |  |  |  |  |  |  | Lower | Upper |
| Step 1(a) | sexo(1) | 1.030 | .092 | 125.138 | 1 | ***.000000*** | ***2.80*** | ***2.34*** | ***3.35*** |
|  | Constant | -2.262 | .074 | 936.238 | 1 | .000 | .104 |  |  |

a Variable(s) entered on step 1: sexo.

**Correlation Matrix**

|  | | Constant | sexo(1) |
| --- | --- | --- | --- |
| Step 1 | Constant | 1.000 | -.803 |
|  | sexo(1) | -.803 | 1.000 |

**Logistic Regression**

**Notes**

| Output Created | | 09-AUG-2005 22:10:50 |
| --- | --- | --- |
| Comments | |  |
| Input | Data | C:\Documents and Settings\ENALDO\My Documents\CALCULOS ESTATISTICOS\TRAB JOSELINA E TAUANE\IC PICANO 280707modifi.sav |
|  | Filter | <none> |
|  | Weight | <none> |
|  | Split File | <none> |
|  | N of Rows in Working Data File | 4042 |
| Missing Value Handling | Definition of Missing | User-defined missing values are treated as missing |
| Syntax | | LOGISTIC REGRESSION CAT50  /METHOD = ENTER dislipid  /CONTRAST (dislipid)=Indicator(1)  /PRINT = CORR CI(95)  /CRITERIA = PIN(.05) POUT(.10) ITERATE(20) CUT(.5) . |
| Resources | Elapsed Time | 0:00:00.16 |

**Case Processing Summary**

| Unweighted Cases(a) | | N | Percent |
| --- | --- | --- | --- |
| Selected Cases | Included in Analysis | 4042 | 100.0 |
|  | Missing Cases | 0 | .0 |
|  | Total | 4042 | 100.0 |
| Unselected Cases | | 0 | .0 |
| Total | | 4042 | 100.0 |

a If weight is in effect, see classification table for the total number of cases.

**Dependent Variable Encoding**

| Original Value | Internal Value |
| --- | --- |
| Coronary Stenosis <50% | 0 |
| Coronary Stenosis >50% | 1 |

**Categorical Variables Codings**

|  | | Frequency | Parameter coding |
| --- | --- | --- | --- |
|  |  |  | (1) |
| DISLIPIDEMIA | falso | 1198 | .000 |
|  | verdadeiro | 2844 | 1.000 |

**Block 0: Beginning Block**

**Classification Table(a,b)**

|  | | | Observed | | | | Predicted |
| --- | --- | --- | --- | --- | --- | --- | --- |
|  |  |  | CAT50 | | Percentage Correct |  | |
|  |  |  | Coronary Stenosis <50% | Coronary Stenosis >50% |  |  | |
| Step 0 | CA50 | Coronary Stenosis <50% | 3411 | 0 | 100.0 |  | |
|  |  | Coronary Stenosis >50% | 631 | 0 | .0 |  | |
|  | Overall Percentage | |  |  | 84.4 |  | |

a Constant is included in the model.

b The cut value is .500

**Variables in the Equation**

|  | | B | S.E. | Wald | df | Sig. | Exp(B) |
| --- | --- | --- | --- | --- | --- | --- | --- |
|
| Step 0 | Constant | -1.687 | .043 | 1516.279 | 1 | .000 | .185 |

**Variables not in the Equation**

|  | | | Score | df | Sig. |
| --- | --- | --- | --- | --- | --- |
| Step 0 | Variables | dislipid(1) | 62.068 | 1 | .000 |
|  | Overall Statistics | | 62.068 | 1 | .000 |

**Block 1: Method = Enter**

**Omnibus Tests of Model Coefficients**

|  | |  | | Chi-square | | df | | Sig. |
| --- | --- | --- | --- | --- | --- | --- | --- | --- |
| Step 1 | Step | 68.163 | 1 | | .000 | |  | |
|  | Block | 68.163 | 1 | | .000 | |  | |
|  | Model | 68.163 | 1 | | .000 | |  | |

**Model Summary**

| Step | -2 Log likelihood | Cox & Snell R Square | Nagelkerke R Square |
| --- | --- | --- | --- |
| 1 | 3433.536(a) | .017 | .029 |

a Estimation terminated at iteration number 5 because parameter estimates changed by less than .001.

**Classification Table(a)**

|  | | | Observed | | | | Predicted |
| --- | --- | --- | --- | --- | --- | --- | --- |
|  |  |  | CA50 | | Percentage Correct |  | |
|  |  |  | Coronary Stenosis <50% | Coronary Stenosis >50% |  |  | |
| Step 1 | CAT50 | Coronary Stenosis <50% | 3411 | 0 | 100.0 |  | |
|  |  | Coronary Stenosis >50% | 631 | 0 | .0 |  | |
|  | Overall Percentage | |  |  | 84.4 |  | |

a The cut value is .500

**Variables in the Equation**

|  | | B | S.E. | Wald | df | Sig. | Exp(B) | 95.0% C.I.for EXP(B) | |
| --- | --- | --- | --- | --- | --- | --- | --- | --- | --- |
|  |  |  |  |  |  |  |  | Lower | Upper |
| Step 1(a) | dyslipid(1) | .872 | .113 | 59.185 | 1 | ***.000000*** | ***2.39*** | ***1.92*** | ***2.99*** |
|  | Constant | -2.353 | .103 | 525.912 | 1 | .000 | .095 |  |  |

a Variable(s) entered on step 1: dislipid.

**Correlation Matrix**

|  | | Constant | dislipid(1) |
| --- | --- | --- | --- |
| Step 1 | Constant | 1.000 | -.905 |
|  | dyslipid(1) | -.905 | 1.000 |

**Logistic Regression**

**Notes**

| Output Created | | 09-AUG-2005 22:12:11 |
| --- | --- | --- |
| Comments | |  |
| Input | Data | C:\Documents and Settings\ENALDO\My Documents\CALCULOS ESTATISTICOS\TRAB JOSELINA E TAUANE\IC PICANO 280707modifi.sav |
|  | Filter | <none> |
|  | Weight | <none> |
|  | Split File | <none> |
|  | N of Rows in Working Data File | 4042 |
| Missing Value Handling | Definition of Missing | User-defined missing values are treated as missing |
| Syntax | | LOGISTIC REGRESSION CAT50  /METHOD = ENTER hiperten  /CONTRAST (hiperten)=Indicator(1)  /PRINT = CORR CI(95)  /CRITERIA = PIN(.05) POUT(.10) ITERATE(20) CUT(.5) . |
| Resources | Elapsed Time | 0:00:00.16 |

**Case Processing Summary**

| Unweighted Cases(a) | | N | Percent |
| --- | --- | --- | --- |
| Selected Cases | Included in Analysis | 4042 | 100.0 |
|  | Missing Cases | 0 | .0 |
|  | Total | 4042 | 100.0 |
| Unselected Cases | | 0 | .0 |
| Total | | 4042 | 100.0 |

a If weight is in effect, see classification table for the total number of cases.

**Dependent Variable Encoding**

| Original Value | Internal Value |
| --- | --- |
| Coronary Stenosis<50% | 0 |
| Coronary Stenosis >50% | 1 |

**Categorical Variables Codings**

|  | | Frequency | Parameter coding |
| --- | --- | --- | --- |
|  |  |  | (1) |
| HIPERTENSÃO | falso | 1720 | .000 |
|  | verdadeiro | 2322 | 1.000 |

**Block 0: Beginning Block**

**Classification Table(a,b)**

|  | | | Observed | | | | Predicted |
| --- | --- | --- | --- | --- | --- | --- | --- |
|  |  |  | CA50 | | Percentage Correct |  | |
|  |  |  | Coronary Stenosis <50% | Coronary Stenosis >50% |  |  | |
| Step 0 | CA50 | Coronary Stenosis <50% | 3411 | 0 | 100.0 |  | |
|  |  | Coronary Stenosis >50% | 631 | 0 | .0 |  | |
|  | Overall Percentage | |  |  | 84.4 |  | |

a Constant is included in the model.

b The cut value is .500

**Variables in the Equation**

|  | | B | S.E. | Wald | df | Sig. | Exp(B) |
| --- | --- | --- | --- | --- | --- | --- | --- |
|
| Step 0 | Constant | -1.687 | .043 | 1516.279 | 1 | .000 | .185 |

**Variables not in the Equation**

|  | | | Score | df | Sig. |
| --- | --- | --- | --- | --- | --- |
| Step 0 | Variables | hiperten(1) | 27.207 | 1 | .000 |
|  | Overall Statistics | | 27.207 | 1 | .000 |

**Block 1: Method = Enter**

**Omnibus Tests of Model Coefficients**

|  | |  | | Chi-square | | df | | Sig. |
| --- | --- | --- | --- | --- | --- | --- | --- | --- |
| Step 1 | Step | 27.797 | 1 | | .000 | |  | |
|  | Block | 27.797 | 1 | | .000 | |  | |
|  | Model | 27.797 | 1 | | .000 | |  | |

**Model Summary**

| Step | -2 Log likelihood | Cox & Snell R Square | Nagelkerke R Square |
| --- | --- | --- | --- |
| 1 | 3473.901(a) | .007 | .012 |

a Estimation terminated at iteration number 5 because parameter estimates changed by less than .001.

**Classification Table(a)**

|  | | | Observed | | | | Predicted |
| --- | --- | --- | --- | --- | --- | --- | --- |
|  |  |  | CAT50 | | Percentage Correct |  | |
|  |  |  | lesao menor de 50 | LESÃO MAIOR DE 50% |  |  | |
| Step 1 | CA50 | Coronary Stenosis 30–50% | 3411 | 0 | 100.0 |  | |
|  |  | LESÃO MAIOR DE 50% | 631 | 0 | .0 |  | |
|  | Overall Percentage | |  |  | 84.4 |  | |

a The cut value is .500

**Variables in the Equation**

|  | | B | S.E. | Wald | df | Sig. | Exp(B) | 95.0% C.I.for EXP(B) | |
| --- | --- | --- | --- | --- | --- | --- | --- | --- | --- |
|  |  |  |  |  |  |  |  | Lower | Upper |
| Step 1(a) | hyperten(1) | .474 | .091 | 26.885 | 1 | ***.000000*** | ***1.61*** | ***1.34*** | ***1.92*** |
|  | Constant | -1.978 | .074 | 718.488 | 1 | .000 | .138 |  |  |

a Variable(s) entered on step 1: hiperten.

**Correlation Matrix**

|  | | Constant | hiperten(1) |
| --- | --- | --- | --- |
| Step 1 | Constant | 1.000 | -.808 |
|  | hiperten(1) | -.808 | 1.000 |

**Logistic Regression**

**Notes**

| Output Created | | 09-AUG-2005 22:13:57 |
| --- | --- | --- |
| Comments | |  |
| Input | Data | C:\Documents and Settings\ENALDO\My Documents\CALCULOS ESTATISTICOS\TRAB JOSELINA E TAUANE\IC PICANO 280707modifi.sav |
|  | Filter | <none> |
|  | Weight | <none> |
|  | Split File | <none> |
|  | N of Rows in Working Data File | 4042 |
| Missing Value Handling | Definition of Missing | User-defined missing values are treated as missing |
| Syntax | | LOGISTIC REGRESSION CAT50  /METHOD = ENTER diabetes  /CONTRAST (diabetes)=Indicator(1)  /PRINT = CORR CI(95)  /CRITERIA = PIN(.05) POUT(.10) ITERATE(20) CUT(.5) . |
| Resources | Elapsed Time | 0:00:00.16 |

**Case Processing Summary**

| Unweighted Cases(a) | | N | Percent |
| --- | --- | --- | --- |
| Selected Cases | Included in Analysis | 4042 | 100.0 |
|  | Missing Cases | 0 | .0 |
|  | Total | 4042 | 100.0 |
| Unselected Cases | | 0 | .0 |
| Total | | 4042 | 100.0 |

a If weight is in effect, see classification table for the total number of cases.

**Dependent Variable Encoding**

| Original Value | Internal Value |
| --- | --- |
| Coronary Stenosis <50% | 0 |
| Coronary Stenosis >50% | 1 |

**Categorical Variables Codings**

|  | | Frequency | Parameter coding |
| --- | --- | --- | --- |
|  |  |  | (1) |
| DIABETES MELLITUS | falso | 3607 | .000 |
|  | verdadeiro | 435 | 1.000 |

**Block 0: Beginning Block**

**Classification Table(a,b)**

|  | | | Observed | | | | Predicted |
| --- | --- | --- | --- | --- | --- | --- | --- |
|  |  |  | CAT50 | | Percentage Correct |  | |
|  |  |  | lesao menor de 50 | LESÃO MAIOR DE 50% |  |  | |
| Step 0 | CA50 | Coronary Stenosis <50% | 3411 | 0 | 100.0 |  | |
|  |  | Coronary Stenosis >50% | 631 | 0 | .0 |  | |
|  | Overall Percentage | |  |  | 84.4 |  | |

a Constant is included in the model.

b The cut value is .500

**Variables in the Equation**

|  | | B | S.E. | Wald | df | Sig. | Exp(B) |
| --- | --- | --- | --- | --- | --- | --- | --- |
|
| Step 0 | Constant | -1.687 | .043 | 1516.279 | 1 | .000 | .185 |

**Variables not in the Equation**

|  | | | Score | df | Sig. |
| --- | --- | --- | --- | --- | --- |
| Step 0 | Variables | diabetes(1) | 77.837 | 1 | .000 |
|  | Overall Statistics | | 77.837 | 1 | .000 |

**Block 1: Method = Enter**

**Omnibus Tests of Model Coefficients**

|  | |  | | Chi-square | | df | | Sig. |
| --- | --- | --- | --- | --- | --- | --- | --- | --- |
| Step 1 | Step | 66.133 | 1 | | .000 | |  | |
|  | Block | 66.133 | 1 | | .000 | |  | |
|  | Model | 66.133 | 1 | | .000 | |  | |

**Model Summary**

| Step | -2 Log likelihood | Cox & Snell R Square | Nagelkerke R Square |
| --- | --- | --- | --- |
| 1 | 3435.566(a) | .016 | .028 |

a Estimation terminated at iteration number 4 because parameter estimates changed by less than .001.

**Classification Table(a)**

|  | | | Observed | | | | Predicted |
| --- | --- | --- | --- | --- | --- | --- | --- |
|  |  |  | CA50 | | Percentage Correct |  | |
|  |  |  | Coronary Stenosis<50% | Coronary Stenosis >50% |  |  | |
| Step 1 | CA50 | Coronary Stenosis<50% | 3411 | 0 | 100.0 |  | |
|  |  | Coronary Stenosis >50% | 631 | 0 | .0 |  | |
|  | Overall Percentage | |  |  | 84.4 |  | |

a The cut value is .500

**Variables in the Equation**

|  | | B | S.E. | Wald | df | Sig. | Exp(B) | 95.0% C.I.for EXP(B) | |
| --- | --- | --- | --- | --- | --- | --- | --- | --- | --- |
|  |  |  |  |  |  |  |  | Lower | Upper |
| Step 1(a) | diabetes(1) | .985 | .115 | 73.249 | 1 | ***.000000*** | ***2.68*** | ***2.14*** | ***3.36*** |
|  | Constant | -1.827 | .048 | 1437.306 | 1 | .000 | .161 |  |  |

a Variable(s) entered on step 1: diabetes.

**Correlation Matrix**

|  | | Constant | diabetes(1) |
| --- | --- | --- | --- |
| Step 1 | Constant | 1.000 | -.419 |
|  | diabetes(1) | -.419 | 1.000 |

**Logistic Regression**

**Notes**

| Output Created | | 09-AUG-2005 22:16:02 |
| --- | --- | --- |
| Comments | |  |
| Input | Data | C:\Documents and Settings\ENALDO\My Documents\CALCULOS ESTATISTICOS\TRAB JOSELINA E TAUANE\IC PICANO 280707modifi.sav |
|  | Filter | <none> |
|  | Weight | <none> |
|  | Split File | <none> |
|  | N of Rows in Working Data File | 4042 |
| Missing Value Handling | Definition of Missing | User-defined missing values are treated as missing |
| Syntax | | LOGISTIC REGRESSION CAT50  /METHOD = ENTER idade  /PRINT = CORR CI(95)  /CRITERIA = PIN(.05) POUT(.10) ITERATE(20) CUT(.5) . |
| Resources | Elapsed Time | 0:00:00.16 |

**Case Processing Summary**

| Unweighted Cases(a) | | N | Percent |
| --- | --- | --- | --- |
| Selected Cases | Included in Analysis | 4039 | 99.9 |
|  | Missing Cases | 3 | .1 |
|  | Total | 4042 | 100.0 |
| Unselected Cases | | 0 | .0 |
| Total | | 4042 | 100.0 |

a If weight is in effect, see classification table for the total number of cases.

**Dependent Variable Encoding**

| Original Value | Internal Value |
| --- | --- |
| Coronary Stenosis 30–50% | 0 |
| LESÃO MAIOR DE 50% | 1 |

**Block 0: Beginning Block**

**Classification Table(a,b)**

|  | | | Observed | | | | Predicted |
| --- | --- | --- | --- | --- | --- | --- | --- |
|  |  |  | CAT50 | | Percentage Correct |  | |
|  |  |  | lesao menor de 50 | LESÃO MAIOR DE 50% |  |  | |
| Step 0 | CAT50 | lesao menor de 50 | 3408 | 0 | 100.0 |  | |
|  |  | LESÃO MAIOR DE 50% | 631 | 0 | .0 |  | |
|  | Overall Percentage | |  |  | 84.4 |  | |

a Constant is included in the model.

b The cut value is .500

**Variables in the Equation**

|  | | B | S.E. | Wald | df | Sig. | Exp(B) |
| --- | --- | --- | --- | --- | --- | --- | --- |
|
| Step 0 | Constant | -1.687 | .043 | 1514.490 | 1 | .000 | .185 |

**Variables not in the Equation**

|  | | | Score | df | Sig. |
| --- | --- | --- | --- | --- | --- |
| Step 0 | Variables | idade | 83.138 | 1 | .000 |
|  | Overall Statistics | | 83.138 | 1 | .000 |

**Block 1: Method = Enter**

**Omnibus Tests of Model Coefficients**

|  | |  | | Chi-square | | df | | Sig. |
| --- | --- | --- | --- | --- | --- | --- | --- | --- |
| Step 1 | Step | 83.619 | 1 | | .000 | |  | |
|  | Block | 83.619 | 1 | | .000 | |  | |
|  | Model | 83.619 | 1 | | .000 | |  | |

**Model Summary**

| Step | -2 Log likelihood | Cox & Snell R Square | Nagelkerke R Square |
| --- | --- | --- | --- |
| 1 | 3417.061(a) | .020 | .035 |

a Estimation terminated at iteration number 5 because parameter estimates changed by less than .001.

**Classification Table(a)**

|  | | | Observed | | | | Predicted |
| --- | --- | --- | --- | --- | --- | --- | --- |
|  |  |  | CA50 | | Percentage Correct |  | |
|  |  |  | Coronary Stenosis <50% | Coronary Stenosis >50% |  |  | |
| Step 1 | CA50 | Coronary Stenosis <50% | 3408 | 0 | 100.0 |  | |
|  |  | Coronary Stenosis >50% | 631 | 0 | .0 |  | |
|  | Overall Percentage | |  |  | 84.4 |  | |

a The cut value is .500

**Variables in the Equation**

|  | | B | S.E. | Wald | df | Sig. | Exp(B) | 95.0% C.I.for EXP(B) | |
| --- | --- | --- | --- | --- | --- | --- | --- | --- | --- |
|  |  |  |  |  |  |  |  | Lower | Upper |
| Step 1(a) | idade | .035 | .004 | 81.267 | 1 | ***.000000*** | ***1.04*** | ***1.03*** | ***1.04*** |
|  | Constant | -3.736 | .237 | 247.941 | 1 | .000 | .024 |  |  |

a Variable(s) entered on step 1: idade.

**Correlation Matrix**

|  | | Constant | idade |
| --- | --- | --- | --- |
| Step 1 | Constant | 1.000 | -.983 |
|  | idade | -.983 | 1.000 |

**Explore**

**Notes**

| Output Created | | 09-AUG-2005 22:28:50 |
| --- | --- | --- |
| Comments | |  |
| Input | Data | C:\Documents and Settings\ENALDO\My Documents\CALCULOS ESTATISTICOS\TRAB JOSELINA E TAUANE\IC PICANO 280707modifi.sav |
|  | Filter | <none> |
|  | Weight | <none> |
|  | Split File | <none> |
|  | N of Rows in Working Data File | 4042 |
| Missing Value Handling | Definition of Missing | User-defined missing values for dependent variables are treated as missing. |
|  | Cases Used | Statistics are based on cases with no missing values for any dependent variable or factor used. |
| Syntax | | EXAMINE  VARIABLES=imc  /PLOT NONE  /PERCENTILES(5,10,25,50,75,90,95) HAVERAGE  /STATISTICS NONE  /MISSING LISTWISE  /NOTOTAL. |
| Resources | Elapsed Time | 0:00:00.08 |

**Case Processing Summary**

|  | Cases | | | | | |
| --- | --- | --- | --- | --- | --- | --- |
|  | Valid | | Missing | | Total | |
|  | N | Percent | N | Percent | N | Percent |
| Índice de Massa Corpórea | 3943 | 97.6% | 99 | 2.4% | 4042 | 100.0% |

**Percentiles**

|  | |  | | | | Percentiles | | | | | | | | | | |
| --- | --- | --- | --- | --- | --- | --- | --- | --- | --- | --- | --- | --- | --- | --- | --- | --- |
|  |  |  |  |  |  | 5 | | 10 | | 25 | | 50 | | 75 | 90 | 95 |
| Weighted Average(Definition 1) | Índice de Massa Corpórea | 21.3000 | 22.4840 | 24.4900 | 26.9800 | | 29.7600 | | 33.0960 | | 35.3640 | |  | | | |
| Tukey's Hinges | Índice de Massa Corpórea |  |  | 24.4900 | 26.9800 | | 29.7600 | |  | |  | |  | | | |

**Frequencies**

**Notes**

| Output Created | | 09-AUG-2005 22:33:21 |
| --- | --- | --- |
| Comments | |  |
| Input | Data | C:\Documents and Settings\ENALDO\My Documents\CALCULOS ESTATISTICOS\TRAB JOSELINA E TAUANE\IC PICANO 280707modifi.sav |
|  | Filter | <none> |
|  | Weight | <none> |
|  | Split File | <none> |
|  | N of Rows in Working Data File | 4042 |
| Missing Value Handling | Definition of Missing | User-defined missing values are treated as missing. |
|  | Cases Used | Statistics are based on all cases with valid data. |
| Syntax | | FREQUENCIES  VARIABLES=IMCCLASS  /ORDER= ANALYSIS . |
| Resources | Elapsed Time | 0:00:00.06 |
|  | Total Values Allowed | 149796 |

**Statistics**

IMCCLASS

| N | Valid | 4042 |
| --- | --- | --- |
|  | Missing | 0 |

**IMCCLASS**

|  | | Frequency | Percent | Valid Percent | Cumulative Percent |
| --- | --- | --- | --- | --- | --- |
| Valid | IMC ATÉ 24.49 | 988 | 24.4 | 24.4 | 24.4 |
|  | DE 24.49 ATÉ 29,76 | 1975 | 48.9 | 48.9 | 73.3 |
|  | DE 29.76 A 33.096 | 586 | 14.5 | 14.5 | 87.8 |
|  | ACIMA DE 33.096 | 493 | 12.2 | 12.2 | 100.0 |
|  | Total | 4042 | 100.0 | 100.0 |  |

**Logistic Regression**

**Notes**

| Output Created | | 09-AUG-2005 22:34:38 |
| --- | --- | --- |
| Comments | |  |
| Input | Data | C:\Documents and Settings\ENALDO\My Documents\CALCULOS ESTATISTICOS\TRAB JOSELINA E TAUANE\IC PICANO 280707modifi.sav |
|  | Filter | <none> |
|  | Weight | <none> |
|  | Split File | <none> |
|  | N of Rows in Working Data File | 4042 |
| Missing Value Handling | Definition of Missing | User-defined missing values are treated as missing |
| Syntax | | LOGISTIC REGRESSION CAT50  /METHOD = ENTER IMCCLASS  /CONTRAST (IMCCLASS)=Indicator  /PRINT = CORR CI(95)  /CRITERIA = PIN(.05) POUT(.10) ITERATE(20) CUT(.5) . |
| Resources | Elapsed Time | 0:00:00.22 |

**Case Processing Summary**

| Unweighted Cases(a) | | N | Percent |
| --- | --- | --- | --- |
| Selected Cases | Included in Analysis | 4042 | 100.0 |
|  | Missing Cases | 0 | .0 |
|  | Total | 4042 | 100.0 |
| Unselected Cases | | 0 | .0 |
| Total | | 4042 | 100.0 |

a If weight is in effect, see classification table for the total number of cases.

**Dependent Variable Encoding**

| Original Value | Internal Value |
| --- | --- |
| Coronary Stenosis <50% | 0 |
| Coronary Stenosis >50% | 1 |

**Categorical Variables Codings**

|  | | Frequency | Parameter coding | | |
| --- | --- | --- | --- | --- | --- |
|  |  |  | (1) | (2) | (3) |
| IMCCLASS | BMI UNTIL 24.49 | 988 | 1.000 | .000 | .000 |
|  | BMI 24.49 TO 29,76 | 1975 | .000 | 1.000 | .000 |
|  | 29.76 TO33.096 | 586 | .000 | .000 | 1.000 |
|  | > 33.096 | 493 | .000 | .000 | .000 |

**Block 0: Beginning Block**

**Classification Table(a,b)**

|  | | | Observed | | | | Predicted |
| --- | --- | --- | --- | --- | --- | --- | --- |
|  |  |  | CA50 | | Percentage Correct |  | |
|  |  |  | Coronary Stenosis <50% | Coronary Stenosis >50% |  |  | |
| Step 0 | CA50 | Coronary Stenosis <50% | 3411 | 0 | 100.0 |  | |
|  |  | Coronary Stenosis >50% | 631 | 0 | .0 |  | |
|  | Overall Percentage | |  |  | 84.4 |  | |

a Constant is included in the model.

b The cut value is .500

**Variables in the Equation**

|  | | B | S.E. | Wald | df | Sig. | Exp(B) |
| --- | --- | --- | --- | --- | --- | --- | --- |
|
| Step 0 | Constant | -1.687 | .043 | 1516.279 | 1 | .000 | .185 |

**Variables not in the Equation**

|  | | | Score | df | Sig. |
| --- | --- | --- | --- | --- | --- |
| Step 0 | Variables | BMICLASS | 6.456 | 3 | .091 |
|  |  | BMICLASS(1) | .051 | 1 | .821 |
|  |  | BMICLASS(2) | 4.957 | 1 | .026 |
|  |  | BMICLASS(3) | 2.753 | 1 | .097 |
|  | Overall Statistics | | 6.456 | 3 | .091 |

**Block 1: Method = Enter**

**Omnibus Tests of Model Coefficients**

|  | |  | | Chi-square | | df | | Sig. |
| --- | --- | --- | --- | --- | --- | --- | --- | --- |
| Step 1 | Step | 6.561 | 3 | | .087 | |  | |
|  | Block | 6.561 | 3 | | .087 | |  | |
|  | Model | 6.561 | 3 | | .087 | |  | |

**Model Summary**

| Step | -2 Log likelihood | Cox & Snell R Square | Nagelkerke R Square |
| --- | --- | --- | --- |
| 1 | 3495.138(a) | .002 | .003 |

a Estimation terminated at iteration number 4 because parameter estimates changed by less than .001.

**Classification Table(a)**

|  | | | Observed | | | | Predicted |
| --- | --- | --- | --- | --- | --- | --- | --- |
|  |  |  | CAT50 | | Percentage Correct |  | |
|  |  |  | Coronary Stenosis<50% | Coronary Stenosis >50% |  |  | |
| Step 1 | CA50 | Coronary Stenosis <50% | 3411 | 0 | 100.0 |  | |
|  |  | Coronary Stenosis >50% | 631 | 0 | .0 |  | |
|  | Overall Percentage | |  |  | 84.4 |  | |

a The cut value is .500

**Variables in the Equation**

|  | | B | S.E. | Wald | df | Sig. | Exp(B) | 95.0% C.I.for EXP(B) | |
| --- | --- | --- | --- | --- | --- | --- | --- | --- | --- |
|  |  |  |  |  |  |  |  | Lower | Upper |
| Step 1(a) | BMICLASS |  |  | 6.434 | 3 | .092 |  |  |  |
|  | BMICLASS(1) | .145 | .158 | .839 | 1 | .360 | 1.156 | .848 | 1.576 |
|  | BMICLASS(2) | .258 | .144 | 3.184 | 1 | .074 | 1.294 | .975 | 1.718 |
|  | BMICLASS(3) | -.024 | .179 | .018 | 1 | .893 | .976 | .687 | 1.387 |
|  | Constant | -1.850 | .131 | 198.090 | 1 | .000 | .157 |  |  |

a Variable(s) entered on step 1: IMCCLASS.

**Correlation Matrix**

|  | | Constant | IMCCLASS(1) | IMCCLASS(2) | IMCCLASS(3) |
| --- | --- | --- | --- | --- | --- |
| Step 1 | Constant | 1.000 | -.830 | -.910 | -.734 |
|  | BMICLASS(1) | -.830 | 1.000 | .755 | .610 |
|  | BMICLASS(2) | -.910 | .755 | 1.000 | .668 |
|  | BMICLASS(3) | -.734 | .610 | .668 | 1.000 |

**Logistic Regression**

**Notes**

| Output Created | | 09-AUG-2005 22:47:39 |
| --- | --- | --- |
| Comments | |  |
| Input | Data | C:\Documents and Settings\ENALDO\My Documents\CALCULOS ESTATISTICOS\TRAB JOSELINA E TAUANE\IC PICANO 280707modifi.sav |
|  | Filter | <none> |
|  | Weight | <none> |
|  | Split File | <none> |
|  | N of Rows in Working Data File | 4042 |
| Missing Value Handling | Definition of Missing | User-defined missing values are treated as missing |
| Syntax | | LOGISTIC REGRESSION CAT50  /METHOD = FSTEP(WALD) altesfor fmg sexo idade dislipid diabetes hiperten  /CONTRAST (altesfor)=Indicator(1) /CONTRAST (fmg)=Indicator /CONTRAST (sexo)=Indicator(1) /CONTRAST (dislipid)=Indicator  (1) /CONTRAST (diabetes)=Indicator(1) /CONTRAST (hiperten)=Indicator(1)  /SAVE = COOK SRESID  /PRINT = GOODFIT CORR CI(95)  /CRITERIA = PIN(.05) POUT(.10) ITERATE(20) CUT(.5) . |
| Resources | Elapsed Time | 0:00:00.56 |
| Variables Created or Modified | COO_1 | Analog of Cook's influence statistics |
|  | SRE_1 | Standard residual |

**Case Processing Summary**

| Unweighted Cases(a) | | N | Percent |
| --- | --- | --- | --- |
| Selected Cases | Included in Analysis | 4039 | 99.9 |
|  | Missing Cases | 3 | .1 |
|  | Total | 4042 | 100.0 |
| Unselected Cases | | 0 | .0 |
| Total | | 4042 | 100.0 |

a If weight is in effect, see classification table for the total number of cases.

**Dependent Variable Encoding**

| Original Value | Internal Value |
| --- | --- |
| Coronary Stenosis <50% | 0 |
| Coronary Stenosis >50% | 1 |

**Categorical Variables Codings**

|  | | Frequency | Parameter coding |
| --- | --- | --- | --- |
|  |  |  | (1) |
| HYPERTENSION | falso | 1719 | .000 |
|  | verdadeiro | 2320 | 1.000 |
| Heart Hate | G1 | 490 | 1.000 |
|  | G2 | 3549 | .000 |
| sex | F | 2139 | .000 |
|  | M | 1900 | 1.000 |
| DYSLIPIDEMIA | falso | 1196 | .000 |
|  | verdadeiro | 2843 | 1.000 |
| DIABETES MELLITUS | falso | 3605 | .000 |
|  | verdadeiro | 434 | 1.000 |
| WM abnormality | falso | 3549 | .000 |
|  | verdadeiro | 490 | 1.000 |

**Block 0: Beginning Block**

**Classification Table(a,b)**

|  | | | Observed | | | | Predicted |
| --- | --- | --- | --- | --- | --- | --- | --- |
|  |  |  | CAT50 | | Percentage Correct |  | |
|  |  |  | Coronary Stenosis 30–50% | LESÃO MAIOR DE 50% |  |  | |
| Step 0 | CA50 | Coronary Stenosis <50% | 3408 | 0 | 100.0 |  | |
|  |  | Coronary Stenosis >50% | 631 | 0 | .0 |  | |
|  | Overall Percentage | |  |  | 84.4 |  | |

a Constant is included in the model.

b The cut value is .500

**Variables in the Equation**

|  | | B | S.E. | Wald | df | Sig. | Exp(B) |
| --- | --- | --- | --- | --- | --- | --- | --- |
|
| Step 0 | Constant | -1.687 | .043 | 1514.490 | 1 | .000 | .185 |

**Variables not in the Equation**

|  | | | Score | df | Sig. |
| --- | --- | --- | --- | --- | --- |
| Step 0 | Variables | WM abnormality (1) | 935.707 | 1 | .000 |
|  |  | fmg(1) | 163.902 | 1 | .000 |
|  |  | sexo(1) | 131.701 | 1 | .000 |
|  |  | idade | 83.138 | 1 | .000 |
|  |  | dislipid(1) | 61.850 | 1 | .000 |
|  |  | diabetes(1) | 78.217 | 1 | .000 |
|  |  | hiperten(1) | 27.249 | 1 | .000 |
|  | Overall Statistics | | 1152.796 | 7 | .000 |

**Block 1: Method = Forward Stepwise (Wald)**

**Omnibus Tests of Model Coefficients**

|  | |  | | Chi-square | | df | | Sig. |
| --- | --- | --- | --- | --- | --- | --- | --- | --- |
| Step 1 | Step | 684.539 | 1 | | .000 | |  | |
|  | Block | 684.539 | 1 | | .000 | |  | |
|  | Model | 684.539 | 1 | | .000 | |  | |
| Step 2 | Step | 106.799 | 1 | | .000 | |  | |
|  | Block | 791.338 | 2 | | .000 | |  | |
|  | Model | 791.338 | 2 | | .000 | |  | |
| Step 3 | Step | 65.782 | 1 | | .000 | |  | |
|  | Block | 857.120 | 3 | | .000 | |  | |
|  | Model | 857.120 | 3 | | .000 | |  | |
| Step 4 | Step | 47.299 | 1 | | .000 | |  | |
|  | Block | 904.419 | 4 | | .000 | |  | |
|  | Model | 904.419 | 4 | | .000 | |  | |
| Step 5 | Step | 34.505 | 1 | | .000 | |  | |
|  | Block | 938.924 | 5 | | .000 | |  | |
|  | Model | 938.924 | 5 | | .000 | |  | |
| Step 6 | Step | 20.520 | 1 | | .000 | |  | |
|  | Block | 959.445 | 6 | | .000 | |  | |
|  | Model | 959.445 | 6 | | .000 | |  | |

**Model Summary**

| Step | -2 Log likelihood | Cox & Snell R Square | Nagelkerke R Square |
| --- | --- | --- | --- |
| 1 | 2816.141(a) | .156 | .269 |
| 2 | 2709.342(a) | .178 | .307 |
| 3 | 2643.560(a) | .191 | .330 |
| 4 | 2596.261(b) | .201 | .346 |
| 5 | 2561.755(b) | .207 | .358 |
| 6 | 2541.235(b) | .211 | .365 |

a Estimation terminated at iteration number 5 because parameter estimates changed by less than .001.

b Estimation terminated at iteration number 6 because parameter estimates changed by less than .001.

**Hosmer and Lemeshow Test**

| Step | Chi-square | df | Sig. |
| --- | --- | --- | --- |
| 1 | .000 | 0 | . |
| 2 | 1.011 | 1 | .315 |
| 3 | 2.023 | 2 | .364 |
| 4 | 13.842 | 8 | .086 |
| 5 | 11.838 | 8 | .159 |
| 6 | 4.215 | 8 | .837 |

**Contingency Table for Hosmer and Lemeshow Test**

|  | | CA50 Coronary Stenosis <50% | | CA50 = Coronary Stenosis >50% | | Total |
| --- | --- | --- | --- | --- | --- | --- |
|  |  | Observed | Expected | Observed | Expected |  |
| Step 1 | 1 | 3225 | 3225.000 | 324 | 324.000 | 3549 |
|  | 2 | 183 | 183.000 | 307 | 307.000 | 490 |
| Step 2 | 1 | 1839 | 1830.939 | 94 | 102.061 | 1933 |
|  | 2 | 1386 | 1394.061 | 230 | 221.939 | 1616 |
|  | 3 | 183 | 183.000 | 307 | 307.000 | 490 |
| Step 3 | 1 | 1642 | 1639.550 | 74 | 76.450 | 1716 |
|  | 2 | 1303 | 1295.999 | 173 | 180.001 | 1476 |
|  | 3 | 280 | 289.450 | 77 | 67.550 | 357 |
|  | 4 | 183 | 183.000 | 307 | 307.000 | 490 |
| Step 4 | 1 | 402 | 394.595 | 4 | 11.405 | 406 |
|  | 2 | 402 | 408.411 | 22 | 15.589 | 424 |
|  | 3 | 401 | 395.979 | 14 | 19.021 | 415 |
|  | 4 | 378 | 380.164 | 26 | 23.836 | 404 |
|  | 5 | 368 | 362.464 | 25 | 30.536 | 393 |
|  | 6 | 351 | 344.751 | 31 | 37.249 | 382 |
|  | 7 | 356 | 362.460 | 55 | 48.540 | 411 |
|  | 8 | 348 | 348.789 | 62 | 61.211 | 410 |
|  | 9 | 278 | 288.202 | 123 | 112.798 | 401 |
|  | 10 | 124 | 122.184 | 269 | 270.816 | 393 |
| Step 5 | 1 | 392 | 392.336 | 8 | 7.664 | 400 |
|  | 2 | 401 | 398.981 | 12 | 14.019 | 413 |
|  | 3 | 387 | 391.399 | 23 | 18.601 | 410 |
|  | 4 | 395 | 386.671 | 15 | 23.329 | 410 |
|  | 5 | 369 | 369.619 | 29 | 28.381 | 398 |
|  | 6 | 376 | 366.840 | 29 | 38.160 | 405 |
|  | 7 | 345 | 339.450 | 42 | 47.550 | 387 |
|  | 8 | 332 | 340.769 | 72 | 63.231 | 404 |
|  | 9 | 279 | 293.300 | 126 | 111.700 | 405 |
|  | 10 | 132 | 128.635 | 275 | 278.365 | 407 |
| Step 6 | 1 | 392 | 390.726 | 6 | 7.274 | 398 |
|  | 2 | 404 | 405.235 | 15 | 13.765 | 419 |
|  | 3 | 386 | 385.495 | 17 | 17.505 | 403 |
|  | 4 | 386 | 384.808 | 21 | 22.192 | 407 |
|  | 5 | 381 | 378.478 | 26 | 28.522 | 407 |
|  | 6 | 363 | 363.131 | 38 | 37.869 | 401 |
|  | 7 | 363 | 354.258 | 40 | 48.742 | 403 |
|  | 8 | 340 | 338.314 | 62 | 63.686 | 402 |
|  | 9 | 276 | 287.574 | 129 | 117.426 | 405 |
|  | 10 | 117 | 119.981 | 277 | 274.019 | 394 |

**Classification Table(a)**

| Observed | | | | | Predicted | | | |
| --- | --- | --- | --- | --- | --- | --- | --- | --- |
| CA50 | | | | | Percentage Correct | | | |
| Coronary Stenosis <50% | | Coronary Stenosis >50% | | |  | | | |
| Step 1 | CA50 | | Coronary Stenosis <50% | 3225 | | 183 | 94.6 |  |
|  |  | | Coronary Stenosis >50% | 324 | | 307 | 48.7 |  |
|  | Overall Percentage | | |  | |  | 87.4 |  |
| Step 2 | CA50 | | Coronary Stenosis <50 | 3323 | | 85 | 97.5 |  |
|  |  | | Coronary Stenosis >50% | 432 | | 199 | 31.5 |  |
|  | Overall Percentage | | |  | |  | 87.2 |  |
| Step 3 | CA50 | | Coronary Stenosis <50% | 3304 | | 104 | 96.9 |  |
|  |  | | Coronary Stenosis >50% | 400 | | 231 | 36.6 |  |
|  | Overall Percentage | | |  | |  | 87.5 |  |
| Step 4 | CA50 | | Coronary Stenosis <50% | 3294 | | 114 | 96.7 |  |
|  |  | | Coronary Stenosis >50% | 389 | | 242 | 38.4 |  |
|  | Overall Percentage | | |  | |  | 87.5 |  |
| Step 5 | CA50 | | Coronary Stenosis <50% | 3300 | | 108 | 96.8 |  |
|  |  | | Coronary Stenosis >50% | 382 | | 249 | 39.5 |  |
|  | Overall Percentage | | |  | |  | 87.9 |  |
| Step 6 | CA50 | | Coronary Stenosis <50% | 3301 | | 107 | 96.9 |  |
|  |  | | Coronary Stenosis >50% | 380 | | 251 | 39.8 |  |
|  | Overall Percentage | | |  | |  | 87.9 |  |

a The cut value is .500

**Variables in the Equation**

|  | | B | S.E. | Wald | df | Sig. | Exp(B) | 95.0% C.I.for EXP(B) | |
| --- | --- | --- | --- | --- | --- | --- | --- | --- | --- |
|  |  |  |  |  |  |  |  | Lower | Upper |
| Step 1(a) | WM abnormality (1) | 2.815 | .110 | 654.048 | 1 | .000 | 16.698 | 13.458 | 20.719 |
|  | Constant | -2.298 | .058 | 1554.705 | 1 | .000 | .100 |  |  |
| Step 2(b) | WM abnormality (1) | 2.828 | .114 | 615.347 | 1 | .000 | 16.904 | 13.520 | 21.136 |
|  | sex (1) | 1.049 | .105 | 100.303 | 1 | .000 | 2.856 | 2.326 | 3.507 |
|  | Constant | -2.887 | .091 | 1001.934 | 1 | .000 | .056 |  |  |
| Step 3(c) | WM abnormality r(1) | 2.724 | .116 | 549.523 | 1 | .000 | 15.235 | 12.132 | 19.131 |
|  | fmg(1) | 1.094 | .131 | 70.210 | 1 | .000 | 2.985 | 2.311 | 3.856 |
|  | sexo(1) | 1.091 | .106 | 105.272 | 1 | .000 | 2.979 | 2.418 | 3.669 |
|  | Constant | -3.066 | .097 | 1004.796 | 1 | .000 | .047 |  |  |
| Step 4(d) | WM abnormality r(1) | 2.663 | .117 | 517.421 | 1 | .000 | 14.335 | 11.396 | 18.032 |
|  | fmg(1) | .999 | .131 | 58.363 | 1 | .000 | 2.716 | 2.102 | 3.510 |
|  | sexo(1) | 1.167 | .108 | 117.234 | 1 | .000 | 3.211 | 2.600 | 3.967 |
|  | idade | .032 | .005 | 46.368 | 1 | .000 | 1.032 | 1.023 | 1.042 |
|  | Constant | -4.921 | .298 | 273.145 | 1 | .000 | .007 |  |  |
| Step 5(e) | WM abnormality (1) | 2.638 | .118 | 501.461 | 1 | .000 | 13.989 | 11.105 | 17.623 |
|  | fmg(1) | .982 | .131 | 55.943 | 1 | .000 | 2.671 | 2.065 | 3.455 |
|  | sexo(1) | 1.182 | .109 | 118.388 | 1 | .000 | 3.259 | 2.635 | 4.032 |
|  | idade | .031 | .005 | 41.629 | 1 | .000 | 1.031 | 1.021 | 1.041 |
|  | dislipid(1) | .739 | .131 | 31.625 | 1 | .000 | 2.094 | 1.619 | 2.710 |
|  | Constant | -5.423 | .320 | 286.577 | 1 | .000 | .004 |  |  |
| Step 6(f) | WM abnormality (1) | 2.606 | .118 | 484.411 | 1 | ***.00000*** | ***13.55*** | ***10.74*** | ***17.09*** |
|  | fmg(1) | .964 | .132 | 53.359 | 1 | ***.00000*** | ***2.62*** | ***2.02*** | ***3.40*** |
|  | sexo(1) | 1.176 | .109 | 116.283 | 1 | ***.00000*** | ***3.24*** | ***2.62*** | ***4.01*** |
|  | idade | .029 | .005 | 37.074 | 1 | ***.00000*** | ***1.03*** | ***1.02*** | ***1.04*** |
|  | dislipid(1) | .731 | .132 | 30.825 | 1 | ***.00000*** | ***2.08*** | ***1.60*** | ***2.69*** |
|  | diabetes(1) | .655 | .141 | 21.554 | 1 | ***.00000*** | ***1.92*** | ***1.46*** | ***2.54*** |
|  | Constant | -5.415 | .323 | 280.811 | 1 | ***.00000*** | .004 |  |  |

a Variable(s) entered on step 1: altesfor.

b Variable(s) entered on step 2: sexo.

c Variable(s) entered on step 3: fmg.

d Variable(s) entered on step 4: idade.

e Variable(s) entered on step 5: dislipid.

f Variable(s) entered on step 6: diabetes.

**Correlation Matrix**

|  | | Constant | WM abnormality (1) | sexo(1) | fmg(1) | idade | dislipid(1) | diabetes(1) |
| --- | --- | --- | --- | --- | --- | --- | --- | --- |
| Step 1 | Constant | 1.000 | -.529 |  |  |  |  |  |
|  | WM abnormality (1) | -.529 | 1.000 |  |  |  |  |  |
| Step 2 | Constant | 1.000 | -.434 | -.763 |  |  |  |  |
|  | WM abnormality (1) | -.434 | 1.000 | .131 |  |  |  |  |
|  | sexo(1) | -.763 | .131 | 1.000 |  |  |  |  |
| Step 3 | Constant | 1.000 | -.401 | -.749 | -.316 |  |  |  |
|  | WM abnormality (1) | -.401 | 1.000 | .123 | -.009 |  |  |  |
|  | fmg(1) | -.316 | -.009 | .093 | 1.000 |  |  |  |
|  | sexo(1) | -.749 | .123 | 1.000 | .093 |  |  |  |
| Step 4 | Constant | 1.000 | -.130 | -.358 | -.023 | -.946 |  |  |
|  | WM abnormality (1) | -.130 | 1.000 | .119 | -.017 | .000 |  |  |
|  | fmg(1) | -.023 | -.017 | .073 | 1.000 | -.083 |  |  |
|  | sexo(1) | -.358 | .119 | 1.000 | .073 | .123 |  |  |
|  | idade | -.946 | .000 | .123 | -.083 | 1.000 |  |  |
| Step 5 | Constant | 1.000 | -.129 | -.360 | -.019 | -.888 | -.323 |  |
|  | WM abnormality r(1) | -.129 | 1.000 | .117 | -.020 | .000 | .027 |  |
|  | fmg(1) | -.019 | -.020 | .069 | 1.000 | -.082 | -.003 |  |
|  | sexo(1) | -.360 | .117 | 1.000 | .069 | .134 | .045 |  |
|  | idade | -.888 | .000 | .134 | -.082 | 1.000 | -.024 |  |
|  | dislipid(1) | -.323 | .027 | .045 | -.003 | -.024 | 1.000 |  |
| Step 6 | Constant | 1.000 | -.125 | -.359 | -.019 | -.887 | -.323 | -.013 |
|  | WM abnormality (1) | -.125 | 1.000 | .115 | -.021 | -.003 | .027 | -.008 |
|  | fmg(1) | -.019 | -.021 | .069 | 1.000 | -.080 | -.004 | -.017 |
|  | sexo(1) | -.359 | .115 | 1.000 | .069 | .134 | .044 | .005 |
|  | idade | -.887 | -.003 | .134 | -.080 | 1.000 | -.020 | -.063 |
|  | dislipid(1) | -.323 | .027 | .044 | -.004 | -.020 | 1.000 | -.004 |
|  | diabetes(1) | -.013 | -.008 | .005 | -.017 | -.063 | -.004 | 1.000 |

**Variables not in the Equation**

|  | | | Score | df | Sig. |
| --- | --- | --- | --- | --- | --- |
| Step 1 | Variables | fmg(1) | 66.073 | 1 | .000 |
|  |  | sexo(1) | 106.181 | 1 | .000 |
|  |  | idade | 41.845 | 1 | .000 |
|  |  | dislipid(1) | 38.801 | 1 | .000 |
|  |  | diabetes(1) | 34.768 | 1 | .000 |
|  |  | hiperten(1) | 21.730 | 1 | .000 |
|  | Overall Statistics | | 274.071 | 6 | .000 |
| Step 2 | Variables | fmg(1) | 72.638 | 1 | .000 |
|  |  | idade | 57.685 | 1 | .000 |
|  |  | dislipid(1) | 39.871 | 1 | .000 |
|  |  | diabetes(1) | 33.304 | 1 | .000 |
|  |  | hiperten(1) | 23.517 | 1 | .000 |
|  | Overall Statistics | | 174.801 | 5 | .000 |
| Step 3 | Variables | idade | 47.132 | 1 | .000 |
|  |  | dislipid(1) | 37.002 | 1 | .000 |
|  |  | diabetes(1) | 29.070 | 1 | .000 |
|  |  | hiperten(1) | 16.313 | 1 | .000 |
|  | Overall Statistics | | 102.723 | 4 | .000 |
| Step 4 | Variables | dislipid(1) | 32.465 | 1 | .000 |
|  |  | diabetes(1) | 22.863 | 1 | .000 |
|  |  | hiperten(1) | 10.306 | 1 | .001 |
|  | Overall Statistics | | 57.876 | 3 | .000 |
| Step 5 | Variables | diabetes(1) | 21.823 | 1 | .000 |
|  |  | hiperten(1) | 5.564 | 1 | .018 |
|  | Overall Statistics | | 25.560 | 2 | .000 |
| Step 6 | Variables | hiperten(1) | 3.735 | 1 | .053 |
|  | Overall Statistics | | 3.735 | 1 | .053 |

**Logistic Regression**

**Notes**

| Output Created | | 09-AUG-2005 22:48:22 |
| --- | --- | --- |
| Comments | |  |
| Input | Data | C:\Documents and Settings\ENALDO\My Documents\CALCULOS ESTATISTICOS\TRAB JOSELINA E TAUANE\IC PICANO 280707modifi.sav |
|  | Filter | <none> |
|  | Weight | <none> |
|  | Split File | <none> |
|  | N of Rows in Working Data File | 4042 |
| Missing Value Handling | Definition of Missing | User-defined missing values are treated as missing |
| Syntax | | LOGISTIC REGRESSION CAT50  /METHOD = BSTEP(LR) altesfor fmg sexo idade dislipid diabetes hiperten  /CONTRAST (altesfor)=Indicator(1) /CONTRAST (fmg)=Indicator /CONTRAST (sexo)=Indicator(1) /CONTRAST (dislipid)=Indicator  (1) /CONTRAST (diabetes)=Indicator(1) /CONTRAST (hiperten)=Indicator(1)  /SAVE = COOK SRESID  /PRINT = GOODFIT CORR CI(95)  /CRITERIA = PIN(.05) POUT(.10) ITERATE(20) CUT(.5) . |
| Resources | Elapsed Time | 0:00:00.75 |
| Variables Created or Modified | COO_2 | Analog of Cook's influence statistics |
|  | SRE_2 | Standard residual |

**Case Processing Summary**

| Unweighted Cases(a) | | N | Percent |
| --- | --- | --- | --- |
| Selected Cases | Included in Analysis | 4039 | 99.9 |
|  | Missing Cases | 3 | .1 |
|  | Total | 4042 | 100.0 |
| Unselected Cases | | 0 | .0 |
| Total | | 4042 | 100.0 |

a If weight is in effect, see classification table for the total number of cases.

**Dependent Variable Encoding**

| Original Value | Internal Value |
| --- | --- |
| Coronary Stenosis <50% | 0 |
| Coronary Stenosis >50% | 1 |

**Categorical Variables Codings**

|  | | Frequency | Parameter coding |
| --- | --- | --- | --- |
|  |  |  | (1) |
| HYPERTENSION | falso | 1719 | .000 |
|  | verdadeiro | 2320 | 1.000 |
| Heart Hate | G1 | 490 | 1.000 |
|  | G2 | 3549 | .000 |
| sex | F | 2139 | .000 |
|  | M | 1900 | 1.000 |
| DYSLIPIDEMIA | falso | 1196 | .000 |
|  | verdadeiro | 2843 | 1.000 |
| DIABETES MELLITUS | falso | 3605 | .000 |
|  | verdadeiro | 434 | 1.000 |
| WM abnormality | falso | 3549 | .000 |
|  | verdadeiro | 490 | 1.000 |

**Block 0: Beginning Block**

**Classification Table(a,b)**

|  | | | Observed | | | | Predicted |
| --- | --- | --- | --- | --- | --- | --- | --- |
|  | | | CAT50 | | Percentage Correct |  | |
|  | | | Coronary Stenosis <50% | Coronary Stenosis >50% |  |  | |
| Step 0 | CA50 | Coronary Stenosis <50% | 3408 | 0 | 100.0 |  | |
|  |  | Coronary Stenosis >50% | 631 | 0 | .0 |  | |
|  | Overall Percentage | |  |  | 84.4 |  | |

a Constant is included in the model.

b The cut value is .500

**Variables in the Equation**

|  | | B | S.E. | Wald | df | Sig. | Exp(B) |
| --- | --- | --- | --- | --- | --- | --- | --- |
|
| Step 0 | Constant | -1.687 | .043 | 1514.490 | 1 | .000 | .185 |

**Variables not in the Equation**

|  | | | Score | df | Sig. |
| --- | --- | --- | --- | --- | --- |
| Step 0 | Variables | WM abnormality (1) | 935.707 | 1 | .000 |
|  |  | fmg(1) | 163.902 | 1 | .000 |
|  |  | sexo(1) | 131.701 | 1 | .000 |
|  |  | idade | 83.138 | 1 | .000 |
|  |  | dislipid(1) | 61.850 | 1 | .000 |
|  |  | diabetes(1) | 78.217 | 1 | .000 |
|  |  | hiperten(1) | 27.249 | 1 | .000 |
|  | Overall Statistics | | 1152.796 | 7 | .000 |

**Block 1: Method = Backward Stepwise (Likelihood Ratio)**

**Omnibus Tests of Model Coefficients**

|  | |  | | Chi-square | | df | | Sig. |
| --- | --- | --- | --- | --- | --- | --- | --- | --- |
| Step 1 | Step | 963.206 | 7 | | .000 | |  | |
|  | Block | 963.206 | 7 | | .000 | |  | |
|  | Model | 963.206 | 7 | | .000 | |  | |

**Model Summary**

| Step | -2 Log likelihood | Cox & Snell R Square | Nagelkerke R Square |
| --- | --- | --- | --- |
| 1 | 2537.474(a) | .212 | .366 |

a Estimation terminated at iteration number 6 because parameter estimates changed by less than .001.

**Hosmer and Lemeshow Test**

| Step | Chi-square | df | Sig. |
| --- | --- | --- | --- |
| 1 | 6.029 | 8 | .644 |

**Contingency Table for Hosmer and Lemeshow Test**

|  | | CAT50 = Coronary Stenosis <50% | | CAT50 = Coronary Stenosis >50% | | Total |
| --- | --- | --- | --- | --- | --- | --- |
|  |  | Observed | Expected | Observed | Expected |  |
| Step 1 | 1 | 401 | 398.669 | 5 | 7.331 | 406 |
|  | 2 | 389 | 390.166 | 14 | 12.834 | 403 |
|  | 3 | 388 | 389.512 | 19 | 17.488 | 407 |
|  | 4 | 385 | 383.011 | 20 | 21.989 | 405 |
|  | 5 | 382 | 378.482 | 25 | 28.518 | 407 |
|  | 6 | 368 | 362.719 | 32 | 37.281 | 400 |
|  | 7 | 360 | 357.301 | 46 | 48.699 | 406 |
|  | 8 | 343 | 340.991 | 63 | 65.009 | 406 |
|  | 9 | 270 | 286.529 | 134 | 117.471 | 404 |
|  | 10 | 122 | 120.620 | 273 | 274.380 | 395 |

**Classification Table(a)**

|  | | | Observed | | | | Predicted |
| --- | --- | --- | --- | --- | --- | --- | --- |
|  |  |  | CAT50 | | Percentage Correct |  | |
|  |  |  | Coronary Stenosis <50% | Coronary Stenosis >50% |  |  | |
| Step 1 | CA50 | Coronary Stenosis <50% | 3302 | 106 | 96.9 |  | |
|  |  | Coronary Stenosis >50% | 383 | 248 | 39.3 |  | |
|  | Overall Percentage | |  |  | 87.9 |  | |

a The cut value is .500

**Variables in the Equation**

|  | | B | S.E. | Wald | df | Sig. | Exp(B) | 95.0% C.I.for EXP(B) | |
| --- | --- | --- | --- | --- | --- | --- | --- | --- | --- |
|  |  |  |  |  |  |  |  | Lower | Upper |
| Step 1(a) | WM abnormality 1) | 2.617 | .119 | 485.775 | 1 | .000 | 13.692 | 10.850 | 17.280 |
|  | fmg(1) | .939 | .133 | 50.075 | 1 | .000 | 2.557 | 1.971 | 3.316 |
|  | sexo(1) | 1.178 | .109 | 116.538 | 1 | .000 | 3.248 | 2.623 | 4.023 |
|  | idade | .028 | .005 | 34.274 | 1 | .000 | 1.029 | 1.019 | 1.038 |
|  | dyslipid(1) | .696 | .133 | 27.411 | 1 | .000 | 2.005 | 1.546 | 2.602 |
|  | diabetes(1) | .626 | .142 | 19.543 | 1 | .000 | 1.871 | 1.417 | 2.469 |
|  | hyperten(1) | .215 | .112 | 3.727 | 1 | .054 | 1.240 | .997 | 1.543 |
|  | Constant | -5.464 | .325 | 281.970 | 1 | .000 | .004 |  |  |

a Variable(s) entered on step 1: altesfor, fmg, sexo, idade, dislipid, diabetes, hiperten.

**Correlation Matrix**

|  | | Constant | altesfor(1) | fmg(1) | sexo(1) | idade | dislipid(1) | diabetes(1) | hiperten(1) |
| --- | --- | --- | --- | --- | --- | --- | --- | --- | --- |
| Step 1 | Constant | 1.000 | -.131 | -.010 | -.359 | -.872 | -.305 | -.005 | -.092 |
|  | WM abnormality (1) | -.131 | 1.000 | -.027 | .116 | -.008 | .020 | -.014 | .066 |
|  | fmg(1) | -.010 | -.027 | 1.000 | .066 | -.070 | .007 | -.009 | -.093 |
|  | sexo(1) | -.359 | .116 | .066 | 1.000 | .133 | .040 | .004 | .018 |
|  | idade | -.872 | -.008 | -.070 | .133 | 1.000 | -.008 | -.052 | -.094 |
|  | dyslipid(1) | -.305 | .020 | .007 | .040 | -.008 | 1.000 | .010 | -.133 |
|  | diabetes(1) | -.005 | -.014 | -.009 | .004 | -.052 | .010 | 1.000 | -.099 |
|  | hyperten(1) | -.092 | .066 | -.093 | .018 | -.094 | -.133 | -.099 | 1.000 |

**Model if Term Removed**

| Variable | | Model Log Likelihood | Change in -2 Log Likelihood | df | Sig. of the Change |
| --- | --- | --- | --- | --- | --- |
| Step 1 | WM abnormality | -1525.130 | 512.785 | 1 | .000 |
|  | fmg | -1292.561 | 47.648 | 1 | .000 |
|  | sex | -1331.384 | 125.293 | 1 | .000 |
|  | age | -1286.087 | 34.699 | 1 | .000 |
|  | dyslipid | -1283.558 | 29.642 | 1 | .000 |
|  | diabetes | -1278.071 | 18.668 | 1 | .000 |
|  | hyperten | -1270.618 | 3.761 | 1 | .052 |

**Logistic Regression**

**Notes**

| Output Created | | 09-AUG-2005 22:49:28 |
| --- | --- | --- |
| Comments | |  |
| Input | Data | C:\Documents and Settings\ENALDO\My Documents\CALCULOS ESTATISTICOS\TRAB JOSELINA E TAUANE\IC PICANO 280707modifi.sav |
|  | Filter | <none> |
|  | Weight | <none> |
|  | Split File | <none> |
|  | N of Rows in Working Data File | 4042 |
| Missing Value Handling | Definition of Missing | User-defined missing values are treated as missing |
| Syntax | | LOGISTIC REGRESSION CAT50  /METHOD = ENTER altesfor fmg sexo idade dislipid diabetes hiperten  /CONTRAST (altesfor)=Indicator(1) /CONTRAST (fmg)=Indicator /CONTRAST (sexo)=Indicator(1) /CONTRAST (dislipid)=Indicator  (1) /CONTRAST (diabetes)=Indicator(1) /CONTRAST (hiperten)=Indicator(1)  /SAVE = COOK SRESID  /PRINT = GOODFIT CORR CI(95)  /CRITERIA = PIN(.05) POUT(.10) ITERATE(20) CUT(.5) . |
| Resources | Elapsed Time | 0:00:00.34 |
| Variables Created or Modified | COO_3 | Analog of Cook's influence statistics |
|  | SRE_3 | Standard residual |

**Case Processing Summary**

| Unweighted Cases(a) | | N | Percent |
| --- | --- | --- | --- |
| Selected Cases | Included in Analysis | 4039 | 99.9 |
|  | Missing Cases | 3 | .1 |
|  | Total | 4042 | 100.0 |
| Unselected Cases | | 0 | .0 |
| Total | | 4042 | 100.0 |

a If weight is in effect, see classification table for the total number of cases.

**Dependent Variable Encoding**

| Original Value | Internal Value |
| --- | --- |
| Coronary Stenosis <50% | 0 |
| Coronary Stenosis >50% | 1 |

**Categorical Variables Codings**

|  | | Frequency | Parameter coding |
| --- | --- | --- | --- |
|  |  |  | (1) |
| HYPERTENSION | falso | 1719 | .000 |
|  | verdadeiro | 2320 | 1.000 |
| Heart Hate | G1 | 490 | 1.000 |
|  | G2 | 3549 | .000 |
| sex | F | 2139 | .000 |
|  | M | 1900 | 1.000 |
| DYSLIPIDEMIA | falso | 1196 | .000 |
|  | verdadeiro | 2843 | 1.000 |
| DIABETES MELLITUS | falso | 3605 | .000 |
|  | verdadeiro | 434 | 1.000 |
| WM abnormality | falso | 3549 | .000 |
|  | verdadeiro | 490 | 1.000 |

**Block 0: Beginning Block**

**Classification Table(a,b)**

|  | | | Observed | | | | Predicted |
| --- | --- | --- | --- | --- | --- | --- | --- |
|  |  |  | CAT50 | | Percentage Correct |  | |
|  |  |  | Coronary Stenosis <50% | Coronary Stenosis >50% |  |  | |
| Step 0 | CA50 | Coronary Stenosis <50% | 3408 | 0 | 100.0 |  | |
|  |  | Coronary Stenosis >50% | 631 | 0 | .0 |  | |
|  | Overall Percentage | |  |  | 84.4 |  | |

a Constant is included in the model.

b The cut value is .500

**Variables in the Equation**

|  | | B | S.E. | Wald | df | Sig. | Exp(B) |
| --- | --- | --- | --- | --- | --- | --- | --- |
|
| Step 0 | Constant | -1.687 | .043 | 1514.490 | 1 | .000 | .185 |

**Variables not in the Equation**

|  | | | Score | df | Sig. |
| --- | --- | --- | --- | --- | --- |
| Step 0 | Variables | altesfor(1) | 935.707 | 1 | .000 |
|  |  | fmg(1) | 163.902 | 1 | .000 |
|  |  | sexo(1) | 131.701 | 1 | .000 |
|  |  | idade | 83.138 | 1 | .000 |
|  |  | dislipid(1) | 61.850 | 1 | .000 |
|  |  | diabetes(1) | 78.217 | 1 | .000 |
|  |  | hiperten(1) | 27.249 | 1 | .000 |
|  | Overall Statistics | | 1152.796 | 7 | .000 |

**Block 1: Method = Enter**

**Omnibus Tests of Model Coefficients**

|  | |  | | Chi-square | | df | | Sig. |
| --- | --- | --- | --- | --- | --- | --- | --- | --- |
| Step 1 | Step | 963.206 | 7 | | .000 | |  | |
|  | Block | 963.206 | 7 | | .000 | |  | |
|  | Model | 963.206 | 7 | | .000 | |  | |

**Model Summary**

| Step | -2 Log likelihood | Cox & Snell R Square | Nagelkerke R Square |
| --- | --- | --- | --- |
| 1 | 2537.474(a) | .212 | .366 |

a Estimation terminated at iteration number 6 because parameter estimates changed by less than .001.

**Hosmer and Lemeshow Test**

| Step | Chi-square | df | Sig. |
| --- | --- | --- | --- |
| 1 | 6.029 | 8 | .644 |

**Contingency Table for Hosmer and Lemeshow Test**

|  | | CA50 Coronary Stenosis <50% | | CA50 Coronary Stenosis >50% | | Total |
| --- | --- | --- | --- | --- | --- | --- |
|  |  | Observed | Expected | Observed | Expected |  |
| Step 1 | 1 | 401 | 398.669 | 5 | 7.331 | 406 |
|  | 2 | 389 | 390.166 | 14 | 12.834 | 403 |
|  | 3 | 388 | 389.512 | 19 | 17.488 | 407 |
|  | 4 | 385 | 383.011 | 20 | 21.989 | 405 |
|  | 5 | 382 | 378.482 | 25 | 28.518 | 407 |
|  | 6 | 368 | 362.719 | 32 | 37.281 | 400 |
|  | 7 | 360 | 357.301 | 46 | 48.699 | 406 |
|  | 8 | 343 | 340.991 | 63 | 65.009 | 406 |
|  | 9 | 270 | 286.529 | 134 | 117.471 | 404 |
|  | 10 | 122 | 120.620 | 273 | 274.380 | 395 |

**Classification Table(a)**

|  | | | Observed | | | | Predicted |
| --- | --- | --- | --- | --- | --- | --- | --- |
|  |  |  | CA50 | | Percentage Correct |  | |
|  |  |  | Coronary Stenosis <50% | Coronary Stenosis >50% |  |  | |
| Step 1 | CA50 | Coronary Stenosis <50% | 3302 | 106 | 96.9 |  | |
|  |  | Coronary Stenosis >50% | 383 | 248 | 39.3 |  | |
|  | Overall Percentage | |  |  | 87.9 |  | |

a The cut value is .500

**Variables in the Equation**

|  | | B | S.E. | Wald | df | Sig. | Exp(B) | 95.0% C.I.for EXP(B) | |
| --- | --- | --- | --- | --- | --- | --- | --- | --- | --- |
|  |  |  |  |  |  |  |  | Lower | Upper |
| Step 1(a) | WM abnormality (1) | 2.617 | .119 | 485.775 | 1 | .000 | 13.692 | 10.850 | 17.280 |
|  | fmg(1) | .939 | .133 | 50.075 | 1 | .000 | 2.557 | 1.971 | 3.316 |
|  | sex(1) | 1.178 | .109 | 116.538 | 1 | .000 | 3.248 | 2.623 | 4.023 |
|  | AGE | .028 | .005 | 34.274 | 1 | .000 | 1.029 | 1.019 | 1.038 |
|  | dyslipid(1) | .696 | .133 | 27.411 | 1 | .000 | 2.005 | 1.546 | 2.602 |
|  | diabetes(1) | .626 | .142 | 19.543 | 1 | .000 | 1.871 | 1.417 | 2.469 |
|  | hyperten(1) | .215 | .112 | 3.727 | 1 | .054 | 1.240 | .997 | 1.543 |
|  | Constant | -5.464 | .325 | 281.970 | 1 | .000 | .004 |  |  |

a Variable(s) entered on step 1: altesfor, fmg, sexo, idade, dislipid, diabetes, hiperten.

**Correlation Matrix**

|  | | Constant | altesfor(1) | fmg(1) | sexo(1) | idade | dislipid(1) | diabetes(1) | hiperten(1) |
| --- | --- | --- | --- | --- | --- | --- | --- | --- | --- |
| Step 1 | Constant | 1.000 | -.131 | -.010 | -.359 | -.872 | -.305 | -.005 | -.092 |
|  | WM abnormality (1) | -.131 | 1.000 | -.027 | .116 | -.008 | .020 | -.014 | .066 |
|  | fmg(1) | -.010 | -.027 | 1.000 | .066 | -.070 | .007 | -.009 | -.093 |
|  | sexo(1) | -.359 | .116 | .066 | 1.000 | .133 | .040 | .004 | .018 |
|  | age | -.872 | -.008 | -.070 | .133 | 1.000 | -.008 | -.052 | -.094 |
|  | dyslipid(1) | -.305 | .020 | .007 | .040 | -.008 | 1.000 | .010 | -.133 |
|  | diabetes(1) | -.005 | -.014 | -.009 | .004 | -.052 | .010 | 1.000 | -.099 |
|  | hyperten(1) | -.092 | .066 | -.093 | .018 | -.094 | -.133 | -.099 | 1.000 |

**Logistic Regression**

**Notes**

| Output Created | | 09-AUG-2005 23:17:38 |
| --- | --- | --- |
| Comments | |  |
| Input | Data | C:\Documents and Settings\ENALDO\My Documents\CALCULOS ESTATISTICOS\TRAB JOSELINA E TAUANE\IC PICANO 280707modifi.sav |
|  | Filter | <none> |
|  | Weight | <none> |
|  | Split File | <none> |
|  | N of Rows in Working Data File | 4042 |
| Missing Value Handling | Definition of Missing | User-defined missing values are treated as missing |
| Syntax | | LOGISTIC REGRESSION CAT50  /METHOD = ENTER altesfor fmg sexo idade dislipid diabetes hiperten tabagism  /CONTRAST (altesfor)=Indicator(1) /CONTRAST (fmg)=Indicator /CONTRAST (sexo)=Indicator(1) /CONTRAST (dislipid)=Indicator  (1) /CONTRAST (diabetes)=Indicator(1) /CONTRAST (hiperten)=Indicator(1) /CONTRAST (tabagism)=Indicator(1)  /SAVE = COOK SRESID  /PRINT = GOODFIT CORR CI(95)  /CRITERIA = PIN(.05) POUT(.10) ITERATE(20) CUT(.5) . |
| Resources | Elapsed Time | 0:00:00.33 |
| Variables Created or Modified | COO_4 | Analog of Cook's influence statistics |
|  | SRE_4 | Standard residual |

**Case Processing Summary**

| Unweighted Cases(a) | | N | Percent |
| --- | --- | --- | --- |
| Selected Cases | Included in Analysis | 4039 | 99.9 |
|  | Missing Cases | 3 | .1 |
|  | Total | 4042 | 100.0 |
| Unselected Cases | | 0 | .0 |
| Total | | 4042 | 100.0 |

a If weight is in effect, see classification table for the total number of cases.

**Dependent Variable Encoding**

| Original Value | Internal Value |
| --- | --- |
| Coronary Stenosis <50% | 0 |
| Coronary Stenosis >50% | 1 |

**Categorical Variables Codings**

|  | | Frequency | Parameter coding |
| --- | --- | --- | --- |
|  |  |  | (1) |
| Cigarrete smoking | falso | 3831 | .000 |
|  | verdadeiro | 208 | 1.000 |
| Heart Hate | G1 | 490 | 1.000 |
|  | G2 | 3549 | .000 |
| sex | F | 2139 | .000 |
|  | M | 1900 | 1.000 |
| DYSLIPIDEMIA | falso | 1196 | .000 |
|  | verdadeiro | 2843 | 1.000 |
| DIABETES MELLITUS | falso | 3605 | .000 |
|  | verdadeiro | 434 | 1.000 |
| HYERTENSÃO | falso | 1719 | .000 |
|  | verdadeiro | 2320 | 1.000 |
| WM abnormality | falso | 3549 | .000 |
|  | verdadeiro | 490 | 1.000 |

**Block 0: Beginning Block**

**Classification Table(a,b)**

|  | | | Observed | | | | Predicted |
| --- | --- | --- | --- | --- | --- | --- | --- |
|  |  |  | CA50 | | Percentage Correct |  | |
|  |  |  | Coronary Stenosis <50% | Coronary Stenosis >50% |  |  | |
| Step 0 | CAT50 | Coronary Stenosis<50% | 3408 | 0 | 100.0 |  | |
|  |  | Coronary Stenosis >50% | 631 | 0 | .0 |  | |
|  | Overall Percentage | |  |  | 84.4 |  | |

a Constant is included in the model.

b The cut value is .500

**Variables in the Equation**

|  | | B | S.E. | Wald | df | Sig. | Exp(B) |
| --- | --- | --- | --- | --- | --- | --- | --- |
|
| Step 0 | Constant | -1.687 | .043 | 1514.490 | 1 | .000 | .185 |

**Variables not in the Equation**

|  | | | Score | df | Sig. |
| --- | --- | --- | --- | --- | --- |
| Step 0 | Variables | WM abnormality (1) | 935.707 | 1 | .000 |
|  |  | fmg(1) | 163.902 | 1 | .000 |
|  |  | sexo(1) | 131.701 | 1 | .000 |
|  |  | idade | 83.138 | 1 | .000 |
|  |  | dislipid(1) | 61.850 | 1 | .000 |
|  |  | diabetes(1) | 78.217 | 1 | .000 |
|  |  | hiperten(1) | 27.249 | 1 | .000 |
|  |  | tabagism(1) | 9.244 | 1 | .002 |
|  | Overall Statistics | | 1153.615 | 8 | .000 |

**Block 1: Method = Enter**

**Omnibus Tests of Model Coefficients**

|  | |  | | Chi-square | | df | | Sig. |
| --- | --- | --- | --- | --- | --- | --- | --- | --- |
| Step 1 | Step | 964.607 | 8 | | .000 | |  | |
|  | Block | 964.607 | 8 | | .000 | |  | |
|  | Model | 964.607 | 8 | | .000 | |  | |

**Model Summary**

| Step | -2 Log likelihood | Cox & Snell R Square | Nagelkerke R Square |
| --- | --- | --- | --- |
| 1 | 2536.073(a) | .212 | .366 |

a Estimation terminated at iteration number 6 because parameter estimates changed by less than .001.

**Hosmer and Lemeshow Test**

| Step | Chi-square | df | Sig. |
| --- | --- | --- | --- |
| 1 | 4.939 | 8 | .764 |

**Contingency Table for Hosmer and Lemeshow Test**

|  | | CAT50 = lesao menor de 50 | | CAT50 = LESÃO MAIOR DE 50% | | Total |
| --- | --- | --- | --- | --- | --- | --- |
|  |  | Observed | Expected | Observed | Expected |  |
| Step 1 | 1 | 400 | 397.744 | 5 | 7.256 | 405 |
|  | 2 | 390 | 391.227 | 14 | 12.773 | 404 |
|  | 3 | 385 | 386.718 | 19 | 17.282 | 404 |
|  | 4 | 383 | 379.372 | 18 | 21.628 | 401 |
|  | 5 | 380 | 376.871 | 25 | 28.129 | 405 |
|  | 6 | 365 | 362.101 | 34 | 36.899 | 399 |
|  | 7 | 359 | 353.262 | 42 | 47.738 | 401 |
|  | 8 | 337 | 339.923 | 67 | 64.077 | 404 |
|  | 9 | 279 | 290.821 | 125 | 113.179 | 404 |
|  | 10 | 130 | 129.961 | 282 | 282.039 | 412 |

**Classification Table(a)**

|  | | | Observed | | | | Predicted |
| --- | --- | --- | --- | --- | --- | --- | --- |
|  |  |  | CAT50 | | Percentage Correct |  | |
|  |  |  | Coronary Stenosis 3<50% | Coronary Stenosis >50% |  |  | |
| Step 1 | CA50 | Coronary Stenosis <50% | 3304 | 104 | 96.9 |  | |
|  |  | Coronary Stenosis >50% | 380 | 251 | 39.8 |  | |
|  | Overall Percentage | |  |  | 88.0 |  | |

a The cut value is .500

**Variables in the Equation**

|  | | B | S.E. | Wald | df | Sig. | Exp(B) | 95.0% C.I.for EXP(B) | |
| --- | --- | --- | --- | --- | --- | --- | --- | --- | --- |
|  |  |  |  |  |  |  |  | Lower | Upper |
| Step 1(a) | WM abnormality (1) | 2.608 | .119 | 480.746 | 1 | .000 | 13.566 | 10.746 | 17.127 |
|  | fmg(1) | .939 | .133 | 50.101 | 1 | .000 | 2.557 | 1.972 | 3.317 |
|  | sex(1) | 1.171 | .109 | 114.818 | 1 | .000 | 3.226 | 2.604 | 3.996 |
|  | age | .029 | .005 | 35.211 | 1 | .000 | 1.029 | 1.019 | 1.039 |
|  | dylipid(1) | .702 | .133 | 27.820 | 1 | .000 | 2.018 | 1.555 | 2.619 |
|  | diabetes(1) | .621 | .142 | 19.212 | 1 | .000 | 1.860 | 1.409 | 2.456 |
|  | hyerten(1) | .216 | .112 | 3.734 | 1 | .053 | 1.241 | .997 | 1.544 |
|  | Cigarrete smoking | .258 | .215 | 1.441 | 1 | .230 | 1.294 | .850 | 1.970 |
|  | Constant | -5.509 | .328 | 281.639 | 1 | .000 | .004 |  |  |

a Variable(s) entered on step 1: altesfor, fmg, sexo, idade, dislipid, diabetes, hiperten, tabagism.

**Correlation Matrix**

|  | | Constant | altesfor(1) | fmg(1) | sexo(1) | idade | dislipid(1) | diabetes(1) | hiperten(1) | tabagism(1) |
| --- | --- | --- | --- | --- | --- | --- | --- | --- | --- | --- |
| Step 1 | Constant | 1.000 | -.123 | -.010 | -.349 | -.873 | -.309 | -.002 | -.090 | -.124 |
|  | WM abnormality (1) | -.123 | 1.000 | -.027 | .119 | -.013 | .017 | -.012 | .065 | -.052 |
|  | fmg(1) | -.010 | -.027 | 1.000 | .067 | -.070 | .008 | -.008 | -.093 | .005 |
|  | sex(1) | -.349 | .119 | .067 | 1.000 | .126 | .040 | .006 | .018 | -.051 |
|  | age | -.873 | -.013 | -.070 | .126 | 1.000 | -.003 | -.054 | -.094 | .098 |
|  | dylipid(1) | -.309 | .017 | .008 | .040 | -.003 | 1.000 | .010 | -.134 | .043 |
|  | diabetes(1) | -.002 | -.012 | -.008 | .006 | -.054 | .010 | 1.000 | -.099 | -.033 |
|  | hyerten(1) | -.090 | .065 | -.093 | .018 | -.094 | -.134 | -.099 | 1.000 | .004 |
|  | Cigarrete smoking (1) | -.124 | -.052 | .005 | -.051 | .098 | .043 | -.033 | .004 | 1.000 |

**Logistic Regression**

**Notes**

| Output Created | | 09-AUG-2005 23:18:01 |
| --- | --- | --- |
| Comments | |  |
| Input | Data | C:\Documents and Settings\ENALDO\My Documents\CALCULOS ESTATISTICOS\TRAB JOSELINA E TAUANE\IC PICANO 280707modifi.sav |
|  | Filter | <none> |
|  | Weight | <none> |
|  | Split File | <none> |
|  | N of Rows in Working Data File | 4042 |
| Missing Value Handling | Definition of Missing | User-defined missing values are treated as missing |
| Syntax | | LOGISTIC REGRESSION CAT50  /METHOD = FSTEP(WALD) altesfor fmg sexo idade dislipid diabetes hiperten tabagism  /CONTRAST (altesfor)=Indicator(1) /CONTRAST (fmg)=Indicator /CONTRAST (sexo)=Indicator(1) /CONTRAST (dislipid)=Indicator  (1) /CONTRAST (diabetes)=Indicator(1) /CONTRAST (hiperten)=Indicator(1) /CONTRAST (tabagism)=Indicator(1)  /SAVE = COOK SRESID  /PRINT = GOODFIT CORR CI(95)  /CRITERIA = PIN(.05) POUT(.10) ITERATE(20) CUT(.5) . |
| Resources | Elapsed Time | 0:00:00.58 |
| Variables Created or Modified | COO_5 | Analog of Cook's influence statistics |
|  | SRE_5 | Standard residual |

**Case Processing Summary**

| Unweighted Cases(a) | | N | Percent |
| --- | --- | --- | --- |
| Selected Cases | Included in Analysis | 4039 | 99.9 |
|  | Missing Cases | 3 | .1 |
|  | Total | 4042 | 100.0 |
| Unselected Cases | | 0 | .0 |
| Total | | 4042 | 100.0 |

a If weight is in effect, see classification table for the total number of cases.

**Dependent Variable Encoding**

| Original Value | Internal Value |
| --- | --- |
| Coronary Stenosis <50% | 0 |
| Coronary Stenosis >50% | 1 |

**Categorical Variables Codings**

|  | | Frequency | Parameter coding |
| --- | --- | --- | --- |
|  |  |  | (1) |
| Cigarrete smoking | falso | 3831 | .000 |
|  | verdadeiro | 208 | 1.000 |
| Heart Hate | G1 | 490 | 1.000 |
|  | G2 | 3549 | .000 |
| sex | F | 2139 | .000 |
|  | M | 1900 | 1.000 |
| DYLIPIDEMIA | falso | 1196 | .000 |
|  | verdadeiro | 2843 | 1.000 |
| DIABETES MELLITUS | falso | 3605 | .000 |
|  | verdadeiro | 434 | 1.000 |
| HYERTENSION | falso | 1719 | .000 |
|  | verdadeiro | 2320 | 1.000 |
| WM abnormality | falso | 3549 | .000 |
|  | verdadeiro | 490 | 1.000 |

**Block 0: Beginning Block**

**Classification Table(a,b)**

|  | | | Observed | | | | Predicted |
| --- | --- | --- | --- | --- | --- | --- | --- |
|  |  |  | CA50 | | Percentage Correct |  | |
|  |  |  | Coronary Stenosis <50% | Coronary Stenosis >50% |  |  | |
| Step 0 | CA50 | Coronary Stenosis >50% | 3408 | 0 | 100.0 |  | |
|  |  | Coronary Stenosis >50% | 631 | 0 | .0 |  | |
|  | Overall Percentage | |  |  | 84.4 |  | |

a Constant is included in the model.

b The cut value is .500

**Variables in the Equation**

|  | | B | S.E. | Wald | df | Sig. | Exp(B) |
| --- | --- | --- | --- | --- | --- | --- | --- |
|
| Step 0 | Constant | -1.687 | .043 | 1514.490 | 1 | .000 | .185 |

**Variables not in the Equation**

|  | | | Score | df | Sig. |
| --- | --- | --- | --- | --- | --- |
| Step 0 | Variables | WM abnormality (1) | 935.707 | 1 | .000 |
|  |  | fmg(1) | 163.902 | 1 | .000 |
|  |  | sexo(1) | 131.701 | 1 | .000 |
|  |  | idade | 83.138 | 1 | .000 |
|  |  | dislipid(1) | 61.850 | 1 | .000 |
|  |  | diabetes(1) | 78.217 | 1 | .000 |
|  |  | hiperten(1) | 27.249 | 1 | .000 |
|  |  | tabagism(1) | 9.244 | 1 | .002 |
|  | Overall Statistics | | 1153.615 | 8 | .000 |

**Block 1: Method = Forward Stepwise (Wald)**

**Omnibus Tests of Model Coefficients**

|  | |  | | Chi-square | | df | | Sig. |
| --- | --- | --- | --- | --- | --- | --- | --- | --- |
| Step 1 | Step | 684.539 | 1 | | .000 | |  | |
|  | Block | 684.539 | 1 | | .000 | |  | |
|  | Model | 684.539 | 1 | | .000 | |  | |
| Step 2 | Step | 106.799 | 1 | | .000 | |  | |
|  | Block | 791.338 | 2 | | .000 | |  | |
|  | Model | 791.338 | 2 | | .000 | |  | |
| Step 3 | Step | 65.782 | 1 | | .000 | |  | |
|  | Block | 857.120 | 3 | | .000 | |  | |
|  | Model | 857.120 | 3 | | .000 | |  | |
| Step 4 | Step | 47.299 | 1 | | .000 | |  | |
|  | Block | 904.419 | 4 | | .000 | |  | |
|  | Model | 904.419 | 4 | | .000 | |  | |
| Step 5 | Step | 34.505 | 1 | | .000 | |  | |
|  | Block | 938.924 | 5 | | .000 | |  | |
|  | Model | 938.924 | 5 | | .000 | |  | |
| Step 6 | Step | 20.520 | 1 | | .000 | |  | |
|  | Block | 959.445 | 6 | | .000 | |  | |
|  | Model | 959.445 | 6 | | .000 | |  | |

**Model Summary**

| Step | -2 Log likelihood | Cox & Snell R Square | Nagelkerke R Square |
| --- | --- | --- | --- |
| 1 | 2816.141(a) | .156 | .269 |
| 2 | 2709.342(a) | .178 | .307 |
| 3 | 2643.560(a) | .191 | .330 |
| 4 | 2596.261(b) | .201 | .346 |
| 5 | 2561.755(b) | .207 | .358 |
| 6 | 2541.235(b) | .211 | .365 |

a Estimation terminated at iteration number 5 because parameter estimates changed by less than .001.

b Estimation terminated at iteration number 6 because parameter estimates changed by less than .001.

**Hosmer and Lemeshow Test**

| Step | Chi-square | df | Sig. |
| --- | --- | --- | --- |
| 1 | .000 | 0 | . |
| 2 | 1.011 | 1 | .315 |
| 3 | 2.023 | 2 | .364 |
| 4 | 13.842 | 8 | .086 |
| 5 | 11.838 | 8 | .159 |
| 6 | 4.215 | 8 | .837 |

**Contingency Table for Hosmer and Lemeshow Test**

|  | | CA50 = Coronary Stenosis <50% | | CA50 Coronary Stenosis >50% | | Total |
| --- | --- | --- | --- | --- | --- | --- |
|  |  | Observed | Expected | Observed | Expected |  |
| Step 1 | 1 | 3225 | 3225.000 | 324 | 324.000 | 3549 |
|  | 2 | 183 | 183.000 | 307 | 307.000 | 490 |
| Step 2 | 1 | 1839 | 1830.939 | 94 | 102.061 | 1933 |
|  | 2 | 1386 | 1394.061 | 230 | 221.939 | 1616 |
|  | 3 | 183 | 183.000 | 307 | 307.000 | 490 |
| Step 3 | 1 | 1642 | 1639.550 | 74 | 76.450 | 1716 |
|  | 2 | 1303 | 1295.999 | 173 | 180.001 | 1476 |
|  | 3 | 280 | 289.450 | 77 | 67.550 | 357 |
|  | 4 | 183 | 183.000 | 307 | 307.000 | 490 |
| Step 4 | 1 | 402 | 394.595 | 4 | 11.405 | 406 |
|  | 2 | 402 | 408.411 | 22 | 15.589 | 424 |
|  | 3 | 401 | 395.979 | 14 | 19.021 | 415 |
|  | 4 | 378 | 380.164 | 26 | 23.836 | 404 |
|  | 5 | 368 | 362.464 | 25 | 30.536 | 393 |
|  | 6 | 351 | 344.751 | 31 | 37.249 | 382 |
|  | 7 | 356 | 362.460 | 55 | 48.540 | 411 |
|  | 8 | 348 | 348.789 | 62 | 61.211 | 410 |
|  | 9 | 278 | 288.202 | 123 | 112.798 | 401 |
|  | 10 | 124 | 122.184 | 269 | 270.816 | 393 |
| Step 5 | 1 | 392 | 392.336 | 8 | 7.664 | 400 |
|  | 2 | 401 | 398.981 | 12 | 14.019 | 413 |
|  | 3 | 387 | 391.399 | 23 | 18.601 | 410 |
|  | 4 | 395 | 386.671 | 15 | 23.329 | 410 |
|  | 5 | 369 | 369.619 | 29 | 28.381 | 398 |
|  | 6 | 376 | 366.840 | 29 | 38.160 | 405 |
|  | 7 | 345 | 339.450 | 42 | 47.550 | 387 |
|  | 8 | 332 | 340.769 | 72 | 63.231 | 404 |
|  | 9 | 279 | 293.300 | 126 | 111.700 | 405 |
|  | 10 | 132 | 128.635 | 275 | 278.365 | 407 |
| Step 6 | 1 | 392 | 390.726 | 6 | 7.274 | 398 |
|  | 2 | 404 | 405.235 | 15 | 13.765 | 419 |
|  | 3 | 386 | 385.495 | 17 | 17.505 | 403 |
|  | 4 | 386 | 384.808 | 21 | 22.192 | 407 |
|  | 5 | 381 | 378.478 | 26 | 28.522 | 407 |
|  | 6 | 363 | 363.131 | 38 | 37.869 | 401 |
|  | 7 | 363 | 354.258 | 40 | 48.742 | 403 |
|  | 8 | 340 | 338.314 | 62 | 63.686 | 402 |
|  | 9 | 276 | 287.574 | 129 | 117.426 | 405 |
|  | 10 | 117 | 119.981 | 277 | 274.019 | 394 |

**Classification Table(a)**

|  | | | Observed | | | | Predicted |
| --- | --- | --- | --- | --- | --- | --- | --- |
|  |  |  | CA50 | | Percentage Correct |  | |
|  |  |  | Coronary Stenosis<50% | Coronary Stenosis >50% |  |  | |
| Step 1 | CA50 | Coronary Stenosis <50% | 3225 | 183 | 94.6 |  | |
|  |  | Coronary Stenosis >50% | 324 | 307 | 48.7 |  | |
|  | Overall Percentage | |  |  | 87.4 |  | |
| Step 2 | CA50 | Coronary Stenosis <50% | 3323 | 85 | 97.5 |  | |
|  |  | Coronary Stenosis >50% | 432 | 199 | 31.5 |  | |
|  | Overall Percentage | |  |  | 87.2 |  | |
| Step 3 | CA50 | Coronary Stenosis <50% | 3304 | 104 | 96.9 |  | |
|  |  | Coronary Stenosis >50% | 400 | 231 | 36.6 |  | |
|  | Overall Percentage | |  |  | 87.5 |  | |
| Step 4 | CA50 | Coronary Stenosis <50% | 3294 | 114 | 96.7 |  | |
|  |  | Coronary Stenosis >50% | 389 | 242 | 38.4 |  | |
|  | Overall Percentage | |  |  | 87.5 |  | |
| Step 5 | CA50 | Coronary Stenosis <50% | 3300 | 108 | 96.8 |  | |
|  |  | Coronary Stenosis >50% | 382 | 249 | 39.5 |  | |
|  | Overall Percentage | |  |  | 87.9 |  | |
| Step 6 | CA50 | Coronary Stenosis <50% | 3301 | 107 | 96.9 |  | |
|  |  | Coronary Stenosis >50% | 380 | 251 | 39.8 |  | |
|  | Overall Percentage | |  |  | 87.9 |  | |

a The cut value is .500

**Variables in the Equation**

|  | | B | S.E. | Wald | df | Sig. | Exp(B) | 95.0% C.I.for EXP(B) | |
| --- | --- | --- | --- | --- | --- | --- | --- | --- | --- |
|  |  |  |  |  |  |  |  | Lower | Upper |
| Step 1(a) | WM abnormality (1) | 2.815 | .110 | 654.048 | 1 | .000 | 16.698 | 13.458 | 20.719 |
|  | Constant | -2.298 | .058 | 1554.705 | 1 | .000 | .100 |  |  |
| Step 2(b) | WM abnormality (1) | 2.828 | .114 | 615.347 | 1 | .000 | 16.904 | 13.520 | 21.136 |
|  | sex(1) | 1.049 | .105 | 100.303 | 1 | .000 | 2.856 | 2.326 | 3.507 |
|  | Constant | -2.887 | .091 | 1001.934 | 1 | .000 | .056 |  |  |
| Step 3(c) | WM abnormality (1) | 2.724 | .116 | 549.523 | 1 | .000 | 15.235 | 12.132 | 19.131 |
|  | fmg(1) | 1.094 | .131 | 70.210 | 1 | .000 | 2.985 | 2.311 | 3.856 |
|  | sexo(1) | 1.091 | .106 | 105.272 | 1 | .000 | 2.979 | 2.418 | 3.669 |
|  | Constant | -3.066 | .097 | 1004.796 | 1 | .000 | .047 |  |  |
| Step 4(d) | WM abnormality (1) | 2.663 | .117 | 517.421 | 1 | .000 | 14.335 | 11.396 | 18.032 |
|  | fmg(1) | .999 | .131 | 58.363 | 1 | .000 | 2.716 | 2.102 | 3.510 |
|  | sexo(1) | 1.167 | .108 | 117.234 | 1 | .000 | 3.211 | 2.600 | 3.967 |
|  | idade | .032 | .005 | 46.368 | 1 | .000 | 1.032 | 1.023 | 1.042 |
|  | Constant | -4.921 | .298 | 273.145 | 1 | .000 | .007 |  |  |
| Step 5(e) | WM abnormality (1) | 2.638 | .118 | 501.461 | 1 | .000 | 13.989 | 11.105 | 17.623 |
|  | fmg(1) | .982 | .131 | 55.943 | 1 | .000 | 2.671 | 2.065 | 3.455 |
|  | sex(1) | 1.182 | .109 | 118.388 | 1 | .000 | 3.259 | 2.635 | 4.032 |
|  | age | .031 | .005 | 41.629 | 1 | .000 | 1.031 | 1.021 | 1.041 |
|  | dYlipid(1) | .739 | .131 | 31.625 | 1 | .000 | 2.094 | 1.619 | 2.710 |
|  | Constant | -5.423 | .320 | 286.577 | 1 | .000 | .004 |  |  |
| Step 6(f) | WM abnormality (1) | 2.606 | .118 | 484.411 | 1 | .000 | 13.551 | 10.744 | 17.092 |
|  | fmg(1) | .964 | .132 | 53.359 | 1 | .000 | 2.623 | 2.025 | 3.397 |
|  | sexo(1) | 1.176 | .109 | 116.283 | 1 | .000 | 3.241 | 2.618 | 4.014 |
|  | idade | .029 | .005 | 37.074 | 1 | .000 | 1.030 | 1.020 | 1.039 |
|  | dYlipid(1) | .731 | .132 | 30.825 | 1 | .000 | 2.078 | 1.605 | 2.690 |
|  | diabetes(1) | .655 | .141 | 21.554 | 1 | .000 | 1.925 | 1.460 | 2.538 |
|  | Constant | -5.415 | .323 | 280.811 | 1 | .000 | .004 |  |  |

a Variable(s) entered on step 1: altesfor.

b Variable(s) entered on step 2: sexo.

c Variable(s) entered on step 3: fmg.

d Variable(s) entered on step 4: idade.

e Variable(s) entered on step 5: dislipid.

f Variable(s) entered on step 6: diabetes.

**Correlation Matrix**

|  | | Constant | altesfor(1) | sex(1) | fmg(1) | age | dylipid(1) | diabetes(1) |
| --- | --- | --- | --- | --- | --- | --- | --- | --- |
| Step 1 | Constant | 1.000 | -.529 |  |  |  |  |  |
|  | WM abnormality (1) | -.529 | 1.000 |  |  |  |  |  |
| Step 2 | Constant | 1.000 | -.434 | -.763 |  |  |  |  |
|  | WM abnormality (1) | -.434 | 1.000 | .131 |  |  |  |  |
|  | sex(1) | -.763 | .131 | 1.000 |  |  |  |  |
| Step 3 | Constant | 1.000 | -.401 | -.749 | -.316 |  |  |  |
|  | altesfor(1) | -.401 | 1.000 | .123 | -.009 |  |  |  |
|  | fmg(1) | -.316 | -.009 | .093 | 1.000 |  |  |  |
|  | sexo(1) | -.749 | .123 | 1.000 | .093 |  |  |  |
| Step 4 | Constant | 1.000 | -.130 | -.358 | -.023 | -.946 |  |  |
|  | altesfor(1) | -.130 | 1.000 | .119 | -.017 | .000 |  |  |
|  | fmg(1) | -.023 | -.017 | .073 | 1.000 | -.083 |  |  |
|  | sex(1) | -.358 | .119 | 1.000 | .073 | .123 |  |  |
|  | idade | -.946 | .000 | .123 | -.083 | 1.000 |  |  |
| Step 5 | Constant | 1.000 | -.129 | -.360 | -.019 | -.888 | -.323 |  |
|  | WM abnormality (1) | -.129 | 1.000 | .117 | -.020 | .000 | .027 |  |
|  | fmg(1) | -.019 | -.020 | .069 | 1.000 | -.082 | -.003 |  |
|  | sex(1) | -.360 | .117 | 1.000 | .069 | .134 | .045 |  |
|  | idade | -.888 | .000 | .134 | -.082 | 1.000 | -.024 |  |
|  | dislipid(1) | -.323 | .027 | .045 | -.003 | -.024 | 1.000 |  |
| Step 6 | Constant | 1.000 | -.125 | -.359 | -.019 | -.887 | -.323 | -.013 |
|  | WM abnormality (1) | -.125 | 1.000 | .115 | -.021 | -.003 | .027 | -.008 |
|  | fmg(1) | -.019 | -.021 | .069 | 1.000 | -.080 | -.004 | -.017 |
|  | sex(1) | -.359 | .115 | 1.000 | .069 | .134 | .044 | .005 |
|  | idade | -.887 | -.003 | .134 | -.080 | 1.000 | -.020 | -.063 |
|  | dylipid(1) | -.323 | .027 | .044 | -.004 | -.020 | 1.000 | -.004 |
|  | diabetes(1) | -.013 | -.008 | .005 | -.017 | -.063 | -.004 | 1.000 |

**Variables not in the Equation**

|  | | | Score | df | Sig. |
| --- | --- | --- | --- | --- | --- |
| Step 1 | Variables | fmg(1) | 66.073 | 1 | .000 |
|  |  | sex(1) | 106.181 | 1 | .000 |
|  |  | idade | 41.845 | 1 | .000 |
|  |  | dislipid(1) | 38.801 | 1 | .000 |
|  |  | diabetes(1) | 34.768 | 1 | .000 |
|  |  | hyerten(1) | 21.730 | 1 | .000 |
|  |  | Cigarrete smoking (1) | 1.617 | 1 | .203 |
|  | Overall Statistics | | 275.083 | 7 | .000 |
| Step 2 | Variables | fmg(1) | 72.638 | 1 | .000 |
|  |  | age | 57.685 | 1 | .000 |
|  |  | dylipid(1) | 39.871 | 1 | .000 |
|  |  | diabetes(1) | 33.304 | 1 | .000 |
|  |  | hyerten(1) | 23.517 | 1 | .000 |
|  |  | Cigarrete smoking (1) | .209 | 1 | .648 |
|  | Overall Statistics | | 175.848 | 6 | .000 |
| Step 3 | Variables | age | 47.132 | 1 | .000 |
|  |  | dylipid(1) | 37.002 | 1 | .000 |
|  |  | diabetes(1) | 29.070 | 1 | .000 |
|  |  | hyerten(1) | 16.313 | 1 | .000 |
|  |  | Cigarrete smoking (1) | .251 | 1 | .616 |
|  | Overall Statistics | | 103.905 | 5 | .000 |
| Step 4 | Variables | dislipid(1) | 32.465 | 1 | .000 |
|  |  | diabetes(1) | 22.863 | 1 | .000 |
|  |  | hypert) | 10.306 | 1 | .001 |
|  |  | Cigarrete smoking (1) | 1.224 | 1 | .269 |
|  | Overall Statistics | | 59.254 | 4 | .000 |
| Step 5 | Variables | diabetes(1) | 21.823 | 1 | .000 |
|  |  | hyerten(1) | 5.564 | 1 | .018 |
|  |  | Cigarrete smoking (1) | 1.785 | 1 | .182 |
|  | Overall Statistics | | 27.030 | 3 | .000 |
| Step 6 | Variables | hyerten(1) | 3.735 | 1 | .053 |
|  |  | Cigarrete smoking (1) | 1.436 | 1 | .231 |
|  | Overall Statistics | | 5.173 | 2 | .075 |

>Error # 1. Command name: _

>The first word in the line is not recognized as an SPSS command.

>This command not executed.
